# A Machine Learning Method to Identify Genetic Variants Potentially Associated With Alzheimer’s Disease

**DOI:** 10.3389/fgene.2021.647436

**Published:** 2021-06-14

**Authors:** Bradley Monk, Andrei Rajkovic, Semar Petrus, Aleks Rajkovic, Terry Gaasterland, Roberto Malinow

**Affiliations:** ^1^Department of Neurosciences, Center for Neural Circuits and Behavior, School of Medicine, University of California, San Diego, San Diego, CA, United States; ^2^Cognitive Science & Psychology IDP, University of California, San Diego, San Diego, CA, United States; ^3^Department of Computer Science, Royal Holloway, University of London, Egham, United Kingdom; ^4^Institute for Genomic Medicine, Scripps Institution of Oceanography, University of California, San Diego, San Diego, CA, United States; ^5^Department of Pathology, Department of Obstetrics, Gynecology and Reproductive Sciences, University of California, San Francisco, San Francisco, CA, United States; ^6^Section of Neurobiology, Division of Biological Sciences, University of California, San Diego, San Diego, CA, United States

**Keywords:** machine learning, neural network, Alzheimer’s, disease, polygenic

## Abstract

There is hope that genomic information will assist prediction, treatment, and understanding of Alzheimer’s disease (AD). Here, using exome data from ∼10,000 individuals, we explore machine learning neural network (NN) methods to estimate the impact of SNPs (i.e., genetic variants) on AD risk. We develop an NN-based method (netSNP) that identifies hundreds of novel potentially protective or at-risk AD-associated SNPs (along with an effect measure); the majority with frequency under 0.01. For case individuals, the number of “protective” (or “at-risk”) netSNP-identified SNPs in their genome correlates positively (or inversely) with their age of AD diagnosis and inversely (or positively) with autopsy neuropathology. The effect measure increases correlations. Simulations suggest our results are not due to genetic linkage, overfitting, or bias introduced by netSNP. These findings suggest that netSNP can identify SNPs associated with AD pathophysiology that may assist with the diagnosis and mechanistic understanding of the disease.

## Introduction

Alzheimer’s disease (AD), the most common form of dementia, is heritable [58–79%, estimated from twin studies (Gatz et al., 2006)], and highly polygenic (Cauwenberghe et al., 2015). Mutations in three genes (*APP*, *PS1*, *PS2*) cause rare forms of the disease [accounting for ∼1% of AD cases (Mendez, 2017)], which shows autosomal dominant transmission with high penetrance and displays an early onset [generally before age 60 (Carmona et al., 2018)]. In the more common form of the disease, late onset AD (LOAD), *APOE* has been established unequivocally as a susceptibility gene (Saunders et al., 1993) with several dozen other genetic loci receiving genetic support (Carmona et al., 2018; Jansen et al., 2019; Kunkle et al., 2019).

The neuropathology of AD is defined by the presence of extracellular senile plaques containing amyloid beta 42 and intracellular neurofibrillary tangles containing hyperphosphorylated tau protein (DeTure and Dickson, 2019). The neuropathological progression of disease has been best described using the Braak staging scheme (I–VI) (Braak et al., 2006). The most important genetic variant in LOAD is the *APOE* ε4 isoform, which predisposes patients to an earlier appearance of AD and a higher Braak score. The role of *APOE* or other identified genetic variants in the pathophysiology of AD is not well understood (Sisodia and George-Hyslop, 2002; Koffie et al., 2012; Karch and Goate, 2014; Shi et al., 2017). Currently available disease biomarkers can be expensive, labor intensive, and do not provide a definitive clinical diagnosis (Gustaw-Rothenberg et al., 2010; Hampel et al., 2018; Jack et al., 2018; Penner et al., 2019). The identification of additional LOAD-linked genetic variants could potentially increase diagnostic accuracy, increase our understanding of the disease, and unmask potential drug targets.

In 2009 two high-powered genome-wide association (GWAS) studies were published that identified, along with *APOE*, several single nucleotide polymorphism (SNPs) loci significantly linked to AD, including SNPs in PICALM, CLU, and CR1 (Harold et al., 2009; Lambert et al., 2009). To continue the search for genetic targets linked to AD, the Alzheimer’s Disease Sequencing Project (ADSP) was established as a collaboration between the National Human Genome Research Institute (NHGRI) and the National Institute on Aging (NIA). As part of this effort, whole-exome sequencing was performed on 5,740 LOAD cases and 5,096 cognitively normal, older individuals, serving as controls (Bis et al., 2018). The overarching goals of this initiative have been to identify novel genomic targets that contribute risk or confer protection toward AD outcomes, and to develop new insights as to why some at-risk individuals do not develop AD. Indeed, data from this project have been used to identify a number of novel SNPs linked to AD (Beecham et al., 2018; Bis et al., 2018; Raghavan et al., 2018; Ma et al., 2019; Patel et al., 2019; Zhang et al., 2019).

Recent studies have presented polygenic risk score (PRS) models for estimating AD risk (Escott-Price et al., 2015, 2017; Desikan et al., 2017; Zhang et al., 2020). In these models, GWAS summary data were used to identify AD-linked genomic variants and to assign each variant a coefficient based on their case-control asymmetries. While PRS models are a powerful method to identify individuals at risk for a disease, we believe they could provide another powerful utility – identifying novel genetic variants that confer AD risk or protection that escape GWAS identification for a number of factors, including rarity and potential interactions (linear and non-linear) with other variants. However, in order to capture these interactions the PRS model needs to be based on individual genotypes (not just GWAS summary data). Here we developed such a model based on individual AD case and control SNP data provided by ADSP. Specifically, artificial neural networks (Sejnowski, 2020) were trained using individual case and control genotypes to estimate polygenic risk. A primary aim of this study was to develop a machine learning-based method (netSNP) that can be used to identify the importance of individual SNPs in a complex polygenic classifier’s decision making process.

netSNP can identify hundreds of AD-linked SNPs, most of which have a low population frequency (<0.01). Supporting the validity of our method are the observations that the number of AD-linked SNPs identified by this method that are harbored by an individual diagnosed with AD is correlated with the age at which that individual’s AD was diagnosed as well as their brain pathology. In particular, the number of risk- (or protection-) linked SNPs an individual harbors correlates negatively (or positively) with the age at which an individual is diagnosed with AD and with their Braak score (i.e., individuals with more risk SNPs had AD at earlier ages and higher Braak scores; individuals with more protective SNPs had AD at later ages and lower Braak scores). Furthermore, scaling the SNPs with a netSNP-derived “importance factor” further increases the correlations. Thus, these correlations provide support for the view that this method correctly identifies AD-linked SNPs and correctly quantifies their importance.

## Results

### Dataset Pipeline, Case:Control Balancing, and SNP Properties

A large variant call format (VCF) datafile [∼200 GB; Alzheimer’s Disease Sequencing Project, ADSP (Beecham et al., 2017)] containing SNP information (i.e., reference or alternate allele for ∼1.4 million SNP sites) on ∼11,000 individuals over the age of 60 (Northern European descent; ∼6,000 diagnosed with AD, and ∼5,000 aged non-AD controls), was organized into a more manageable file (∼2 GB; N.B.: a VCF datafile contains mainly zeros – indicating reference alleles – since >95% of minor allele frequencies are <0.01) permitting rapid queries regarding SNP content for any individual (see section “Materials and Methods”). The minor frequency allele (MFA) and reference allele count were determined at each SNP locus, separately for case and control groups. The Fisher’s exact test was used to quantify the probability (FishP) that the observed case/control minor allele asymmetry could be due to chance.

The ADSP dataset consists of SNP information originating from 24 cohort groups (Beecham et al., 2017; Crane et al., 2017; Naj et al., 2018). We initially tested if a neural net (NN) classifier could be trained (Moller, 1993), with SNPs as features (50 SNPs with the lowest FishP values; 50 features were chosen as this was computationally tractable; see section “Materials and Methods”), to identify from which cohort an individual originated. Indeed, the classifier could identify cohort identity for each individual with ∼50% accuracy, much above the 4% expected by chance (Supplementary Figures 1, 2). This was of concern, because given the large case:control imbalance in many cohorts (see Supplementary Figure 2), the classifier could use cohort information to help identify patient AD status. Thus, the SNPs would indicate something about the cohort (e.g., platform- or probe-specific aspects of cohorts) rather than the disease. To avoid this potentially confounding issue, we balanced cohorts. In short, (a) only cohorts with at least 20% of the cohort being cases or controls were used; and (b) the same number of case and control individuals from each cohort was used in training sets (see Supplementary Figure 2 and “Materials and Methods” for details).

**FIGURE 1:**
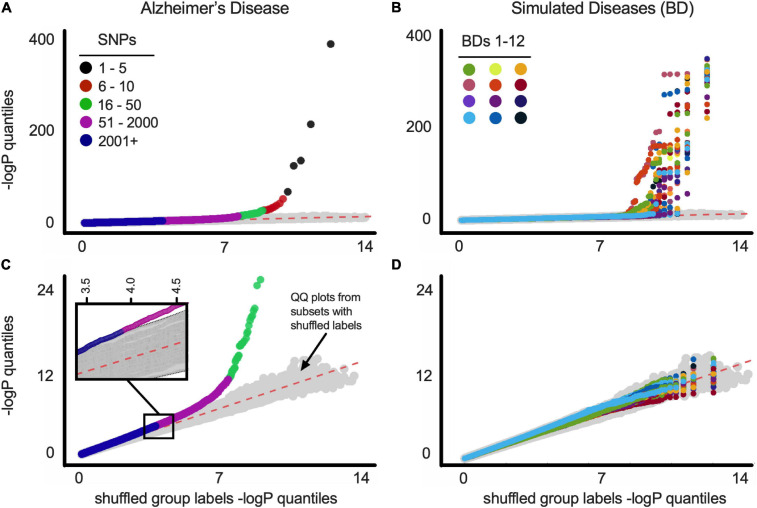
**(A)** Q-Q plots of balanced ADSP dataset for Alzheimer’s disease. Gray symbols (here and below) represent Q-Q plot of 100 random versus random distributions (i.e., chance). See text for details. SNP order [based on −*log*(P)] is indicated by colors (see legend). **(B)** Q-Q plots of balanced ADSP dataset for 12 constructed (simulated) diseases (BDs). Each BD is represented by a different color. **(C)** Same as above with SNPs from APOE-residing chromosome 19 removed before *p*-value quantiles were computed. Inset is a blow up of the indicated region, showing magenta SNPs fall outside the 100 random versus random distributions (gray symbols). SNP order [based on −*log*(P)] is indicated by colors (see legend). **(D)** Same as above with SNPs from BDgene-residing chromosome removed before *p*-value quantiles were computed. Plot as in B is shown for all BD1-33 in Supplementary Figure 12.

A quantile-quantile (Q-Q) plot of the -log(FishP) values of a balanced dataset plotted against a similar computation of the same dataset with shuffled case-control labels shows that most of the case-control minor allele asymmetries across the 1.4 million SNP loci can be explained by chance (i.e., lie on the x = y line; Figures 1A–D). For comparison we plotted 100 Q-Q plots, wherein -log(FishP) values from one shuffled dataset was plotted against -log(FishP) values from another shuffled dataset (Figures 1A–D, gray symbols). For the AD population, in a few SNPs from *APOE* and (its physically close linkage partner) TOMM40 genes (Yu et al., 2007; Guerreiro and Hardy, 2012), the observed distribution of reference allele (Ref) and MFA in the case and control populations was far (orders of magnitude) from what can be accounted for by chance (Figures 1A,C).

To address the possibility that artifacts can account for SNPs off the x = y line (e.g., SPNs being linked to *APOE*, rather than to AD), we constructed 33 separate simulated diseases (“bad diseases,” BDs) using all ADSP individuals (cf., Bulik-Sullivan et al., 2015). Each BD was based on an existing gene (BDgene) that has two SNPs with frequencies in our population very close to *APOE*ε4 (0.147, E4-like) and *APOE*ε2 (0.076, E2-like); see Table 1, MAF (minor allele frequency) columns. Individuals with the BDgene genotype (i.e., having E4-like or E2-like SNPs) in the ADSP dataset were randomly ascribed to have BDs based on control/case odds ratio of *APOE*ε4 (0.30) and *APOE*ε2 (2.41) for AD. Individuals without BDgene SNPs were randomly assigned based on the odds ratio of individuals without *APOE*ε4 or ε2 (i.e., *APOE*ε33 = 0.89). FishP values were computed for each SNP from true (AD) and simulated (BDs) diseases from balanced data sets, and Q-Q plots were generated (Figures 1B,D). Plots including all SNPs showed many with FishP values outside what could be accounted for by chance for both AD and BDs (Figures 1A,B). However, if SNPs from chromosome 19 (where *APOE* resides) or the chromosome with BDgene were removed, only SNPs for AD could not be accounted for by chance (Figures 1C,D). This result is consistent with the view previously observed that AD is a highly polygenic disorder (Cauwenberghe et al., 2015; Escott-Price et al., 2017) as there was a considerable asymmetry in MAF between case and control populations for over 2,000 SNPs (see Figure 1D). While artifacts related to data stratification can account for this behavior in Q-Q plots (Lander and Schork, 1994; Slatkin, 2007), cohort balancing and our simulations argue against such artifacts for our dataset, and support the existence of a large number of SNPs linked to AD, consistent with previous results (Cauwenberghe et al., 2015; Escott-Price et al., 2017).

**TABLE 1:** netSNP identified tSNPs with greatest absolute average *CVt* when APOE locus variants were not excluded from the training set.

tSNPs predicted to confer most protection against AD						tSNPs predicted to confer the most risk for AD					
Chr	Pos	Gene	*mCVt*	FishP	MAF	Chr	Pos	Gene	*mCVt*	FishP	MAF
5	612,536	CEP72	−0.243	5.7E-03	0.002	11	10,327,875	ADM	0.289	4.1E-08	0.008
6	1,390,303	FOXF2	−0.182	1.4E-02	0.003	19	45,411,941	APOE ε4*	0.261	3.4E-111	0.135
4	110,638,764	PLA2G12A	−0.178	2.8E-02	0.002	7	23,213,734	KLHL7	0.217	5.1E-03	0.003
1	16,890,642	NBPF1	−0.174	3.3E-02	0.002	9	130,439,029	STXBP1	0.207	1.9E-02	0.001
11	1,017,294	MUC6	−0.157	1.4E-07	0.01	20	37,258,198	ARHGAP40	0.203	1.1E-02	0.002
1	40,961,395	ZFP69	−0.156	4.8E-04	0.002	15	41,862,356	TYRO3	0.197	8.4E-18	0.018
15	50,154,563	ATP8B4	−0.156	2.3E-02	0.004	6	146,276,263	SHPRH	0.195	6.6E-03	0.002
19	52,793,834	ZNF766	−0.155	2.8E-02	0.002	1	228,879,367	RHOU	0.195	6.4E-03	0.004
15	64,017,685	HERC1	−0.152	3.5E-03	0.004	9	131,398,647	WDR34	0.19	7.0E-03	0.002
19	45,412,079	APOE ε2*	−0.152	7.1E-38	0.079	19	52,497,235	ZNF615	0.188	3.3E-02	0.003
16	8,740,006	METTL22	−0.15	2.1E-03	0.002	12	85,450,243	LRRIQ1	0.188	1.0E-02	0.006
9	139,396,933	NOTCH1	−0.143	3.3E-02	0.004	15	25,963,545	ATP10A	0.185	1.2E-02	0.002
19	18,561,473	ELL	−0.137	7.3E-03	0.008	12	108,011,971	BTBD11	0.183	3.8E-03	0.007
11	57,467,411	ZDHHC5	−0.133	3.5E-03	0.002	9	107,533,232	NIPSNAP3B	0.181	1.1E-02	0.003
9	100,372,648	TSTD2	−0.131	2.2E-02	0.003	1	8,420,270	RERE	0.18	3.0E-02	0.004
1	65,120,426	CACHD1	−0.131	1.0E-02	0.002	8	10,480,495	RP1L1	0.179	3.1E-02	0.003
12	69,113,184	NUP107	−0.126	5.6E-03	0.006	4	5,682,993	EVC2	0.178	2.2E-02	0.004
5	145,508,644	LARS	−0.126	1.2E-02	0.006	5	140,530,973	PCDHB6	0.178	1.4E-02	0.002
7	6,561,105	GRID2IP	−0.125	2.9E-04	0.002	6	30,712,298	IER3	0.177	3.3E-03	0.007
19	43,268,061	PSG8	−0.125	2.9E-02	0.004	15	50,264,839	ATP8B4	0.176	1.1E-02	0.008
11	47,264,353	ACP2	−0.125	1.4E-03	0.004	16	3,604,305	NLRC3	0.176	2.7E-02	0.002
6	7,405,242	RIOK1	−0.124	1.5E-02	0.003	22	46,780,446	CELSR1	0.174	1.9E-02	0.003
3	146,167,089	PLSCR2	−0.123	2.3E-02	0.003	19	39,103,307	MAP4K1	0.173	1.9E-02	0.001
16	30,775,522	RNF40	−0.123	2.9E-02	0.006	1	89,579,827	GBP2	0.173	2.5E-02	0.005
9	139,008,644	C9orf69	−0.121	2.9E-03	0.003	12	50,500,080	GPD1	0.173	2.6E-03	0.002
*Rows 26:1000 available online*							*Rows 26:1000 available online*				

* Previously published AD-linked gene

* Previously published AD-linked gene

### NN Construction and Performance

**FIGURE 2:**
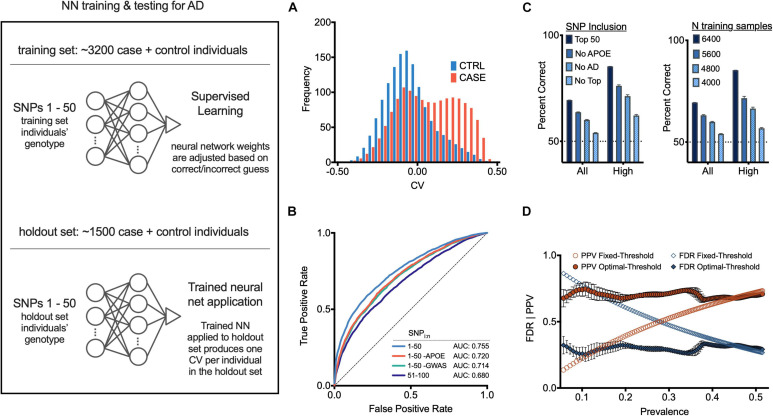
Neural net prediction of case and control individuals. (Left) General neural net protocol and architecture; see text for details. **(A)** Histogram of neural net confidence value (CV) output for case (red) and control (blue) holdout set individuals. **(B)** Receiver operator characteristic (ROC) curves for indicated SNP sets as features. **(C)** (Left) Neural net accuracy for indicated populations using indicated SNP sets as features. “All CV” and “High abs(CV)” indicate inclusion of populations with 100% or top 30% abs(CV) values in accuracy calculations. Error bars, SEM. (Right) Neural net accuracy with indicated size training set; two samples (chromosomes) per individual. **(D)** False discovery rate (FDR) and positive predictive value (PPV) at each corresponding *x*-axis case prevalence, using a fixed CV threshold (set to 0) or optimal operating point classification threshold (see section “Materials and Methods”). Additional measures are in Supplementary Figures 9–11.

Once the cohorts were balanced, we calculated the FishP values for SNPs from a “*training set*” composed of 3,200 randomly chosen individuals (case + controls; equal number of each) and used the 50 SNPs with the lowest FishP values in the training set to train an NN classifier to predict if an individual was a case or control (Figure 2, left; see section “Materials and Methods”). Briefly (Demuth et al., 2014), an artificial NN was trained to classify cases vs. controls using genotypes (for 50 SNPs) of individuals in the training set. The NN was initialized with random weights connecting each node, so the initial prediction *y* was random (each *y* was a real number scaled between −0.5, the control label, and +0.5, the case label). This prediction, also known as a classifier value (CV), was evaluated against the true label (case or control) using a loss function, and the network weights were updated using an optimization function. Throughout training the optimizer adjusts NN weights, working to minimize the loss function. Training concluded when the NN weights were considered optimal (within the constraints of the stopping criteria and cross validation; see section “Materials and Methods”), at which point the NN weights remain fixed. Thus, additional input to the NN would yield CV predictions, but would *not* change network weights or alter the model in any regard.

After the NN was trained as described above, it was applied to the 50 SNPs of each individual from the “*holdout set*” (1,500 individuals randomly chosen who were not included in the *training set*), providing a CV for each. Overall these CVs correlated well with actual AD status of each individual (case, red; control, blue; Figure 2A and Supplementary Figure 4). Using a classification threshold of zero (such that any positive CV was predicted as *case*, and any negative CV was predicted as *control*), the classifier accuracy was 67.3% (SD = 0.3%, see section “Materials and Methods”). The NN performance with 50 SNPs was significantly better than what could be achieved using only SNPs from the *APOE* locus (62.2%). It also performed better than a logistic regression model using the same 50 SNPs (64.2%, *p* < 10e-20, McNemar Test). When only considering individuals with CVs closer to −0.5 or +0.5, the accuracy of the NN increased. For individuals with CVs in the outer quartiles, prediction accuracy was 76.4% (SD = 0.5%); for those with a CV ranked in the upper 12.5% and lower 12.5% quantiles, the classification accuracy was 82.6% (SD = 0.6%) (see Figure 2C).

We next trained an NN using a set of 50 SNPs (a) not containing *APOE* gene SNPs, or (b) not containing the 22 previously published AD-associated SNPs (Carmona et al., 2018), or (c) with the 51–100 lowest FishP values. The resulting accuracy and receiver operator characteristic (ROC) curves, which provide a measure of the sensitivity and specificity of a method (Koen et al., 2016), were all above chance in predicting AD status of an individual (Figures 2B,C). Reducing the size of the training set reduced the accuracy in a roughly linear fashion (Figure 2C), suggesting that the NN accuracy did not asymptote at 3,200 individuals, and that gathering SNP information from more individuals would increase NN accuracy. The area under the ROC curve (AUC) for our NN model with 50 SNPs was 0.755. Further analysis of NN hyperparameters such as the number of SNPs, which SNPs were employed, NN architecture, etc., may improve NN performance; we note that producing an optimal NN was not the primary goal of this study. Other methods, such as PCA (Jolliffe, 1986; Selzam et al., 2018), or Random Forest (Goldstein et al., 2011) analyses were not examined.

Due to the cohort counterbalancing requirement (see above), the prevalence of AD in our training set was 0.5. Since disease prevalence in most populations will almost certainly be lower than 0.5, we quantified signal detection metrics for a range of disease prevalence rates from 0.05 to 0.5 (0.05 is the approximate AD prevalence at age 75), using the optimal operating point (OOP) for each respective base rate (see section “Materials and Methods”). Using the OOP, the false discovery rate was largely independent of prevalence for values from ∼0.05 to 0.5 (Figure 2D). Similarly, the same optimal threshold maintained a largely constant positive predictive rate (Figure 2D). Thus, computing an NN with training data composed of an equal number of cases and controls can be used despite a low disease prevalence.

### netSNP Description and Application

While NNs can perform well in solving complex problems, determining the importance of different NN input features (in this case, different SNPs) is difficult to assess. With this in mind, we developed a method (netSNP) using a modification of the standard NN protocol, aimed to assess the impact of any SNP on conferring AD risk or protection. Specifically, we derived a quantitative measure for the impact of an SNP on the output of an NN.

netSNP is a modification of the *Permutation Importance* method used in machine learning (Altmann et al., 2010; Molnar, 2021), which we have adapted for use with polygenic models. In general Permutation Importance is used to address the question “What variables have the biggest impact on the predictions of a trained neural network classifier?” Permutation Importance computations are performed after a model has already been fitted, and works using a basic strategy: a single predictor variable is modified in the input data, leaving all the other predictor variables unchanged, and examining how this affects classifier performance. This procedure is then repeated, one variable at a time, for all the predictor variables used in the model. This permits one to determine the relative effect of each predictor variable. The netSNP method uses a similar strategy. For a specific SNP, netSNP addresses this question “if this SNP is artificially made homozygous for the MFA, what impact does it have on the classifier output?” If the average CV shifts to the right (e.g., goes from 0.1 to 0.3) when a SNP is set to homozygous for the MFA, netSNP deems this SNP to confer risk. If the average CV shifts to the left (e.g., goes from 0.1 to −0.2) when a SNP is set to homozygous for the MFA, netSNP deems this SNP to confer protection.

**FIGURE 3:**
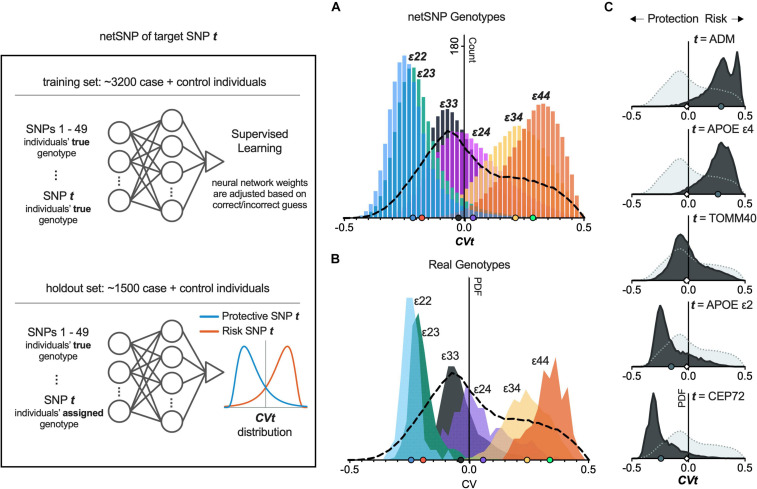
netSNP accurately reproduces CV values for APOE genotypes and identifies potential AD-risk and AD-protective SNPs. (Left) Diagram of netSNP method; details in text. SNP t assignment, APOE genotype for A; and homozygous MFA for C. **(A)** netSNP-generated CVt values for all holdout set individuals (all APOE genotypes) with their genotype artificially assigned to indicated genotype. The dashed line indicates distribution of CV values for all holdout set individuals with correct genotype. **(B)** Frequency distribution of CV values for holdout set subsets containing only individuals with indicated APOE genotype. Dashed line as above. **(C)** Example netSNP-generated CV distributions for all holdout individuals with true genotype (light gray) or CVt with indicated target SNPs (dark gray) assigned alt/alt; symbols on *X*-axis: mean CVt (mCVt) values. Note that mCVt value for TOMM40 SNP is close to zero, indicating that it perturbs NN output little (i.e., provides little additional information) when APOE SNPs are used in training. CVt distributions for 50 tSNPs shifting CV most to the left and 50 tSNPs shifting CV most to the right are shown in Supplementary Figure 5. tSNPs having potentially a protective effect on individuals with APOE4 are shown in Supplementary Figure 5, Supplementary Table 2, and online.

To demonstrate the netSNP method on a specific example, we used netSNP to compute the impact of the *APOE* genotype on AD risk. From a balanced dataset, we randomly chose a training set composed of 3,200 cases+controls (which contained individuals with all *APOE* genomic variants; i.e., *APOE* ε22, ε23, ε24, ε33, ε34, and ε44). This set was used to train an NN (which we call NNε) to identify cases or controls based on their top 50 SNPs (see section “Materials and Methods” and Figure 3 left panel, top). After this training session, NNε was not modified in the subsequent analysis of the *APOE* genomic variants. We then applied NNε to a holdout set of 1,500 individuals (Figure 3, left panel, bottom), producing 1,500 CV outputs with a distribution shown in Figure 3A (dashed line; this is used as a *baseline* for comparisons). We next reasoned that the impact of a specific *APOE* genotype on NNε predictions could be assessed by artificially modifying the *APOE* genotype of every holdout set individual to that specific *APOE* genotype. For instance, to assess the impact of the ε22 genotype, we artificially assigned every holdout set individual the *APOE* ε22 genotype (keeping non-APOE genotypes of each individual unaltered). After applying NNε to these modified genotypes, the distribution of CV outputs was strongly shifted leftward compared to the *baseline* distribution (Figure 3A, compare blue distribution to dashed line). Alternatively, if we assigned all holdout set individuals the ε44 genotype, the CV distribution shifted significantly rightward from *baseline* (Figure 3A, compare orange distribution to dashed line). Falling between the ε22 and ε44 distributions were the CV distributions when NNε was applied to holdout set individuals assigned either the ε23, or ε33, or ε24, or ε34 genotype (Figure 3A).

We next performed a critical test of netSNP: to determine if the above (i.e., the colored CV distributions in Figure 3A) corresponded to distributions when NNε was applied for individuals who did have distinct *APOE* genotypes. To test for this, we created holdout sets with individuals with only one *APOE* genotype (i.e., one holdout set included only *APOE* ε22 individuals, another holdout set only *APOE* ε23 individuals, etc.). We then used NNε to compute CVs for individuals in each of these holdout sets (using the true genotypes for each individual, for 50 SNPs). The resulting CV distribution for true *APOE* genotype holdout sets moved from left to right as *APOE* changed from ε22 to ε44 (Figure 3B), closely matching the CV distributions from above, where *APOE* status was assigned to all individuals in the holdout set (compare Figures 3A,B). This result suggests that netSNP can accurately assess the impact of individual SNPs on a classifier output.

Since *APOE* SNPs are known to significantly impact AD risk, this result also suggests that the netSNP method could be used to estimate the impact of many different target SNPs of interest (which we call ***tSNPs***) on AD risk. We achieved this for each ***tSNP*** by performing the following procedure (analogous to the procedure used to test the impact of APOE genotypes above; see Figure 3, left panel): From a balanced dataset, we randomly chose a training set composed of 3,200 cases+controls. This set was used to train an NN (which we call NNt) to identify cases or controls based on their true genotypes for 50 SNPs (the top 49 SNPs based on FishP value, and the ***tSNP*** of interest). Then we constructed a holdout set of 1,500 individuals, and applied NNt on each individual, using the same 50 SNPs used in training, and using the true genotypes of each individual. This produced 1,500 baseline CV values. Finally we constructed a holdout set of 1,500 individuals, and used the same 50 SNPs, using their true genotypes for each individual for 49 SNPs, but the ***tSNP*** was set to be homozygous for the ***tSNP*** MFA. We then applied the NNt producing 1,500 CV values (which we call ***CVt***) which can be plotted in a frequency distribution (Figure 3, bottom). We repeated this procedure for many ***tSNP****s* (see section “Materials and Methods”; Figure 3C shows ***CVt*** distributions for several ***tSNP****s*). Intuitively, we reasoned that if an SNP had an effect on AD risk, then when evaluated as a ***tSNP***, the ***CVt*** distribution would be shifted compared to the baseline CV distribution – shifts to the left would indicate the MFA SNP is AD-protective (Figure 3, left panel, “Protective SNP ***t***” distribution); shifts to the right would indicate the MFA SNP incurs AD risk; the larger the shift, the greater the impact on AD. We test this proposal below.

We used netSNP to test 4,000 individual SNPs as ***tSNP***s; we chose those SNPs with the 4,000 lowest FishP values. Each ***tSNP*** was evaluated 20 times (see section “Materials and Methods”) from which a mean ***CVt*** (***mCVt***) is computed over all holdout set individuals for all 20 runs. Evaluating *APOE*ε4 as a ***tSNP*** with netSNP resulted in a ***CV*_ε4_** distribution that was shifted to the right (Figure 3C, 2nd from top; same as Figure 3A, green), as expected. Surprisingly, the MFA of an adrenomedullin (*ADM*) SNP shifted the CV distribution more to the right than *APOE*ε4 (***mCV*_ε4_** = 0.26 ± 0.001; ***mCV*_*ADM*_** = 0.29 ± 0.001). Also, a number of SNPs shifted ***NNt*** output CVs more to the left than *APOE*ε2 (e.g., ***mCV*_ε2_** = −0.15 ± 0.001; ***mCV*_*CEP72*_** = −0.24 ± 0.001; see Table 1 for ***tSNP***s with the most extreme ***mCVt***. Thus netSNP appears to identify a number of SNPs that can considerably shift NN output CV, potentially identifying SNPs that confer AD protection (shifting CV to the left) and AD risk (shifting CV to the right).

To exclude the artifactual possibility that netSNP was dependent on *APOE*, we repeated the netSNP method with *APOE* (and *TOMM40*) SNPs excluded from the 49 SNPs with the lowest FishP values as features in training ***NNt*** (although *APOE* was tested as a target ***tSNP***). Results were very similar to the above, with hundreds of ***tSNP***s shifting CV to the right (potentially AD risk SNPs) and hundreds of ***tSNP***s shifting CV to the left (potentially AD protective SNPs; Supplementary Table 1). In general, this method provides a quantitative measure of the impact (as indicated by ***mCVt*** values) of specific SNPs on NN output, and potentially (see below) the effect of such SNPs on developing AD.

### NN and CV as Predictors of AD and Its Pathophysiology

**FIGURE 4:**
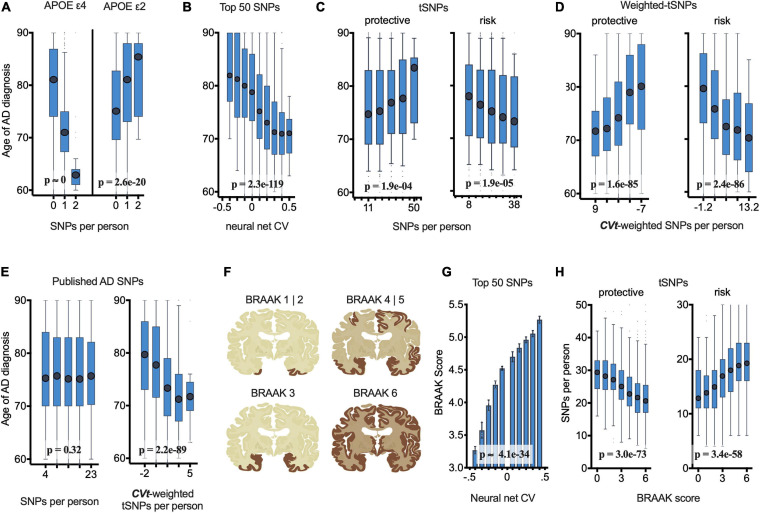
netSNP validation: Number of netSNP-identified tSNPs and netSNP-CVt-weighted tSNPs correlate with age of AD diagnosis of all case individuals (*N = 4752*) and AD pathology (case and control receiving autopsy, *N = 2700*). (See Supplementary Figure 8 for results excluding APOE and TOMM40 in netSNP training matrices; results are similar; conclusions are the same.) **(A)** Age of AD diagnosis plotted against the number of APOE e4 (or APOE e2) SNPs per person. Here and below: boxplot *X*-axis values indicate mean value of boxed group; *p*-values based on general linear model analysis of variance. **(B)** Age of AD diagnosis plotted versus 50 SNP neural net CV. **(C)** Age of AD diagnosis plotted versus number of netSNP-identified AD-protective, left, or AD-risk tSNPs. **(D)** Age of AD diagnosis plotted versus netSNP-CVt-weighted number of AD-protective (left) and AD-risk (right) tSNPs. **(E)** Age of AD diagnosis plotted versus number (left) or netSNP-CVt-weighted number (right) of previously published AD-linked SNPs, excluding those in APOE/TOMM40. n.s., not significant. **(F)** Diagram of human brain with affected regions for indicated Braak scores. **(G)** Neural net CV plotted versus Braak score. **(H)** netSNP-identified AD-protective (left) and AD-risk (right) tSNPs per person plotted versus Braak score.

While CV values (computed with or without APOE as an NN feature) predict well the likelihood of an individual being diagnosed with AD (Supplementary Figure 4), we aimed to determine if CV values correlate with the pathophysiology underlying AD. We reasoned that individuals diagnosed with AD at an earlier age may have a more aggressive form of the disease, which could be a consequence of their genetics, and this might be detected by more positive CV values; equivalently, AD diagnosis at an older age may correlate with less aggressive AD pathophysiology, and may have more negative CV values. This reasoning is supported by previous findings with *APOE* genotypes (Corder et al., 1993), which we found to also be true in our dataset (Figure 4A). Linear regression fitting shows that, for case individuals, as their *APOE*ε2 allele count increases, so does their observed disease onset age [*F*(2,4750) = 86, β (slope; indicating number of years per ε2 allele) = 3.8, *p* < 2.6e-20; general linear model, see section “Materials and Methods”]; conversely, the number of *APOE*ε4 alleles reduces the age of AD diagnosis [*F*(2,4750) = 1910, β = −8.4, *p* < 1e-300]. With this reasoning in mind, we tested and found that the age at which cases were diagnosed with AD could be predicted by their CV values [as computed in section “NN Construction and Performance”; more positive CV for younger age of AD diagnosis, *F*(2,4752) = 571, β = −27, *p* < 2.3e-119; Figure 4B]. Furthermore, an individual’s CV was positively correlated with Braak score, for case individuals receiving autopsy [*F*(2,2025) = 154, β = 1.8, *p* < 4.1e-34; Figure 4G]. These effects were also highly significant if APOE was not included in the NN calculation of CV [CV vs. age, *F*(2,4752) = 422, β = −22.4, *p* = 5.3e-90; CV vs. Braak, *F*(2,2025) = 59, β = 1.7, *p* = 1.8e-14]. These findings support the view that the NN output value CV, as described above in section “NN Construction and Performance,” is related to the pathophysiology of AD.

### netSNP as Predictor of AD-Linked *tSNP*s and AD Pathophysiology

We next tested if netSNP can identify AD-linked SNPs and can quantify their impact on the likelihood of developing AD. We considered a set of ***tSNP***s for which their computed ***mCVt*** values were significantly (*p* < 0.05) outside the range of ***mCVt*** values generated by randomly choosing target SNPs from the set of all 1.4 × 10^6^ ADSP SNPs (see section “Materials and Methods”). This resulted in 851 ***tSNP***s with ***mCVt*** < 0 (provisionally indicated “AD-protective ***tSNP***s”) and 672 ***tSNP***s with ***mCVt*** > 0 (“AD-risk ***tSNP***s”), the majority (64%) with MAF under 0.01. Only some of the previously published AD-linked SNPs (which we exclude from the subsequent validation analysis) are in these sets (see Table 1). Using a general linear model, we found that the number of “AD-protective ***tSNPs***” harbored by each case individual correlated positively with their age of AD diagnosis [*F*(2,4750) = 13.9, β = 0.072, *p* < 1.9e-4; Figure 4C], while the number of “AD-risk ***tSNPs***” they harbored correlated inversely with age of AD diagnosis [*F*(2,4750) = 18.2, β = −0.11, *p* < 1.9e-05; Figure 4C]. Providing ***tSNPs*** with a ***CVt*** weight increased the positive correlation between ***CVt***-weighted “AD protective ***tSNPs***” [*F*(2,4750) = 400, β = 22, *p* < 1.6e-85], or the negative correlation between ***CVt***-weighted “AD risk ***tSNPs***” [*F*(2,4750) = 404, β = −25, *p* < 2.4e-86; Figure 4D] and age of AD diagnosis. Interestingly, the number of previously published AD risk SNPs (excluding *APOE* and *TOMM40* SNPs) per individual did not correlate with age of AD diagnosis (*p* = 0.32; Figure 4E). However, if netSNP is used to calculate ***CVt*** for each of these SNPs, the number of ***CVt***-weighted SNPs did correlate inversely with age of AD diagnosis [*F*(2,4750) = 419, β = −22, *p* < 2.2e-89; Figure 4E], supporting the view that ***CVt*** provides a quantitative measure of the impact of an SNP on AD pathophysiology. We were concerned that the netSNP method may ascribe ***CVt*** values to SNPs based on genetic linkage to *APOE*ε2 or ε4, therefore we performed simulations using BD populations (see section “Materials and Methods”: *netSNP Validation Simulations*). These simulations support the view that the netSNP method is not choosing AD “protective” and AD “at-risk” SNPs based on genetic linkage or some other bias introduced in the netSNP procedure.

We also examined the relation of netSNP-identified ***tSNPs*** to the Braak scores that individuals (cases and controls) received during autopsy. The number of netSNP-identified “AD protective ***tSNPs***” harbored per person displayed a negative correlation with Braak scores [*F*(2,2698) = 349, β = −0.08, *p* < 3.0e-73; Figure 4H], while the number of netSNP-identified “AD risk ***tSNPs***” harbored per person displayed a positive correlation with Braak scores [*F*(2,2698) = 272, β = 0.09, *p* < 3.4e-58; Figure 4H]. These significant correlations, and the effect of providing ***CVt*** weights, were obtained if *APOE* and *TOMM40* SNPs were (Figure 4) or were not (Supplementary Figure 8) included in the training matrix, indicating that the observed correlations were not driven by *APOE* (or SNPs in linkage disequilibrium with *APOE*; see Supplementary Figure 13 and “Materials and Methods”).

## Discussion

Here we applied a standard and modified neural network tool to a large LOAD dataset and examined the association of SNPs to AD. We found that a standard NN trained with 50 SNPs can identify an individual’s cohort identity above chance; thus data were subsequently analyzed using only cohorts that were case:control balanced. Comparing Q-Q plots for AD and simulated constructed diseases (based on real genes with SNPs that have true population frequency as *APOE*ε2 and ε4) supports previous suggestions (Escott-Price et al., 2015, 2017) that there exist considerably more SNPs than the ∼20 previously identified as AD-associated.

An NN trained with 50 SNPs can predict dataset cases with accuracy greater (albeit, slightly) than if using only *APOE* SNPs genotypes, or a basic logistic regression model. NN accuracy was related approximately linearly with training set size, suggesting increasing dataset size will increase NN accuracy. NN accuracy was above chance if an NN was trained without (a) *APOE* SNPs, or (b) previously published AD-linked SNPs, or (c) 50 SNPs displaying the greatest case control asymmetry. These findings further support the view (Escott-Price et al., 2015, 2017) that more than the previously identified SNPs contain information regarding AD.

We developed netSNP, which investigated the impact of specific SNPs on NN output. In netSNP, once an NN was trained, the holdout set genotype was artificially assigned at a single (or multiple) target SNP(s); in the general case the target SNP was assigned as homozygous to the minor frequency allele; the effect of the artificially introduced genotype was reflected by how much the NN output value was modified. netSNP recapitulated well the effect of different *APOE* genotypes on NN output. netSNP identified several hundred SNPs with weight values (i.e., ***mCVt***) significantly outside values produced by randomly chosen SNPs. Some netSNP-identified SNPs had more extreme weight values than *APOE*ε2 or ε4. Notably, FishP values of SNPs with extreme ***mCVt*** values were not low in general, likely because too few individuals carry these SNPs. Yet their impact on NN output was large, possibly by leveraging non-linear interactions embedded in an NN. Notably, ADM (containing an SNP with the largest ***mCVt*** value despite a MAF = 0.009) was elevated in AD brains (Ferrero et al., 2017), contributed to age-related memory loss in mice (Larrayoz et al., 2017), was elevated in aging human brains (Larrayoz et al., 2017), and had been proposed as a novel drug target for AD (Ferrero et al., 2018). We also examined ABHD17A, as it relates to findings indicating that reduced function of this enzyme increases synaptic PSD-95 levels (Jeyifous et al., 2016; Yokoi et al., 2016), which protect synapses from beta amyloid (Malinow, *unpublished observation*). netSNP predicted that an *ABHD17A* SNP was protective for individuals with *APOE*ε4 (see Supplementary Table 2). Indeed, we found that ε4 carrier case individuals with this *ABHD17A* SNP received an AD diagnosis almost 6 years later than such individuals without this SNP [76.6 years (*N* = 19) vs. 70.8 years (*N* = 1831), *p* < 0.0001; *t*-test], which is consistent with this SNP being protective against AD in *APOE*ε4 carriers. These findings support the view that netSNP can identify AD-relevant SNPs.

To validate netSNP we considered variables not used in any netSNP computations: age of an individual’s AD diagnosis (cf., Mars et al., 2020) and Braak score. The number of netSNP-identified “AD-protective SNPs” harbored by an individual correlated significantly with the age an individual was diagnosed with AD and inversely with Braak score; while the number of netSNP-identified “AD-risk SNPs” harbored by an individual correlated significantly inversely with the age an individual was diagnosed with AD and positively with Braak score. Scaling each netSNP-identified SNP with ***CVt*** increased the significance of these correlations. Notably, applying netSNP-derived ***CVt*** weights to previously reported AD SNPs (each thought to have a small effect on AD pathophysiology) converted their correlation to age of diagnosis from not significant to significant, suggesting that netSNP can accurately assess small-effect SNPs. The correlations examined in this validation test hold if *APOE* or *TOMM40* are not used in the training step of netSNP, indicating that the netSNP-identified SNPs as well as the netSNP-generated ***CVt*** weights are not dependent on a bias imposed by *APOE* SNPs (or SNPs in linkage disequilibrium with APOE, Supplementary Figure 13) in netSNP. Further validation of netSNP and net-SNP-identified SNPs suggested to be “protective” or “at-risk” in this study will require tests using an independent AD dataset as well as biological experimentation.

Our data suggest the set, as a whole, of netSNP-identified SNPs are highly predictive of AD age of onset and physiological severity, and their relative importance may be indicated by the netSNP-derived ***mCVt*** weight. The netSNP-identified SNPs would each, on average, be expected to have a small impact on the disease (on average ∼1/200 that of *APOE*ε4; but see above for *ABHD17A* SNP). Insight into AD provided by such small-effect SNPs will require computational methods that can analyze disease and biochemical pathways from large groups of genes. Such tools may be aided by incorporation of ***mCVt*** values.

In general, our findings suggest that netSNP may be useful in identifying pathophysiologically relevant genes in AD; it may be equally applicable to other conditions. It will be important to test these methods on a completely independent AD dataset with similar ethnic make-up (and compare those results with results in this study), as well as AD datasets with different ethnic backgrounds, for this method to be generally applicable to the multicultural nature of the United States and world population (Martin et al., 2019).

## Materials and Methods

### Alzheimer’s Disease Sequencing Project Dataset

The dataset used in these analyses was generously provided by the Alzheimer’s Disease Sequencing Project (ADSP), and has been previously described in detail in other manuscripts (Harold et al., 2009; Raghavan et al., 2018) and online at niagads.org. To summarize, individuals in this dataset were from well-characterized cohorts, including ∼6,000 individuals diagnosed with late-onset Alzheimer’s disease (mean age of diagnosis: 75.4) and ∼5,000 elderly controls without dementia (mean age: 86.1, at the date of last visit to AD practitioner). Whole-exome sequencing data for each individual went through a quality-control “cleaning” process by two independent sources (Baylor and Broad Institutes), and was provided in variant call format (.vcf); genotype data was accompanied by several phenotypic and qualitative metrics (e.g., each individual’s sex, age, race, cohort, etc.). For ∼28% of individuals an autopsy was performed and their Braak staging score was reported (Braak et al., 2006). Data are available for download upon administrative approval from the NIA Genetics of Alzheimer’s Disease Storage Site (NIAGADS).

### VCF Data Compression

Raw SNP data were passed through an automated preprocessing pipeline that involved reducing the dataset size by ∼100-fold using sparse matrices and annotating SNPs of interest. The raw data were downloaded to a secure local hard drive as VCFs. VCFs were formatted as a matrix with rows being loci and columns being samples. This matrix was converted into a structure like an adjacency list. Sample IDs were replaced with seven-digit IDs. Flags passed through the VCFs were converted to numeric flags. Counts of homozygous and heterozygous samples, as well as the sample names and genotypes were recorded per locus. The dataset was binned into three bins according to the following criteria: first, if the genotype was heterozygous (noted as 1), or homozygous (noted as 2) for the alternate allele. The second, if the genotype was homozygous for the reference allele (noted as 0). Third, if there was missing data for that sample (noted as −1). The combination of the bins and information contained within makes the ∼100-fold compression conversion a lossless process. The resulting matrices were relatively small and thus easier to query/manipulate than VCFs.

### General Data Processing

Unless otherwise stated, data processing and analyses were conducted using MATLAB scientific computing software (Mathworks, 2020a,b). A compressed version of the data (as described in the section above) was imported into the MATLAB workspace. The data were then prepared for machine learning by splitting the data into training and holdout datasets. As the data were split, an attempt was made to balance cases and controls from each cohort. Cohorts that had too few cases or controls (<20% of each other; or fewer than 20 individuals) were omitted (see Supplementary Figure 2). After splitting and counterbalancing, a Fisher’s exact test was performed for each SNP to assign a *p*-value to the case:control asymmetries. SNPs were then sorted, ascending, by *p*-value.

### Artificial Neural Network Classification

In most instances, the model training matrix (feature matrix) consisted of individual genotypes for the 50 top SNPs after sorting SNPs by the training group’s *Fisher’s exact test p*-value. The rows and columns of this feature matrix represented individuals and SNPs, respectively, with each cell indicating whether a person was a homozygous reference, heterozygous, or homozygous alternate (see Supplementary Figure 3).

For polygenic classification we used a multilayer pattern recognition neural network (Mathworks, 2020a). This feed-forward neural net architecture can be trained to predict target classes (i.e., “labels” or “conditions” like case/control) based on a set of training features (Demuth et al., 2014). Labels for pattern recognition networks in a binary classification problem consist of a vector of 0 s and 1 s, where a 0 represents the negative condition (i.e., control), while a 1 represents the positive condition (i.e., case). In our formulation a pattern recognition network includes the following parameterization:

patternnet(nLayers,fTrain,fPerf)

where *nLayers* is the row vector of length n, representing the number of hidden layers; each nth value specifies the number of neurons in a given layer (e.g., [50,10] would have two hidden layers of 50 neurons and 10 neurons, respectively). *fTrain* specifies the network training function (e.g., BFGS Quasi-Newton). *fPerf* specifies the performance function (e.g., cross-entropy).

We used a scaled conjugate gradient (SCG) training function for the polygenic classification task (*fTrain* = SCG). The SCG network training function updates network weights and bias values using conjugate gradient backpropagation, and can be used to train any network with derivatives for weight, input, and transfer functions (Moller, 1993). With regard to network training speed, SCG is significantly faster than other conjugate gradient methods, because it does not require line searches during each machine learning iteration (∼0.1 core hours per training session). Parameterization of the training function involves:

*fTrain* (*maxEpochs, minGrad, maxFails, WtSigma, Lambda*)

where *maxEpochs* is the maximum number of epochs to train (e.g., 1000), *minGradient* is the minimum performance gradient (e.g., 1e-6), *maxFails* is the maximum validation failures allowed (e.g., 10), *WtSigma* is the change in weight for second derivative approximation (e.g., 5.0e-5), and *Lambda* regulates the indefiniteness of the Hessian (e.g., 5.0e-7). Unless otherwise noted, the model was implemented in the MATLAB (*Mathworks – Deep Learning Toolbox*) scientific programming environment and parameterized with the following values:

$$\begin{array}{l} patternnet(nLayers=(50,\;10),fTrain=“SCG”,\\ \;fPerf=“cross-entropy”)\\ SCG(maxEpochs=1000,\;minGrad=1e-6,\;maxFails=10,\\ WtSigma=5e-5,\;Lambda=5e-7)\\ \;cross-entropy(reg=0.1,\;norm=(-0.5,0.5)). \end{array}$$

The last steps involve preparing the data for network training: (1) individuals are randomly split into a training, validation, or holdout group; (2) a Fisher’s exact test is used to compute the *p*-value associated with the case:control asymmetry in the training set at each variant locus; (3) the list of SNPs are sorted, ascending by *p*-value; and (4), some number of SNPs (e.g., the top 50) are selected for generating an individual-by-SNP matrix, where each cell contains the genotype of a given person at a given SNP locus. Finally, with the feature matrices prepared, and the model fully parameterized, neural net training can commence:

net=t⁢r⁢a⁢i⁢n⁢(p⁢a⁢t⁢t⁢e⁢r⁢n⁢n⁢e⁢t,X⁢t,Y⁢t,X⁢v,Y⁢v)

Again, *patternnet* represents the parameterized model (and all instructions for model training), *Xt* and *Xv* represent the individual-by-SNP feature matrix for the training and validation groups, respectively, and *Yt* and *Yv* are binary arrays indicating whether each person is a case or control (i.e., the condition labels). The model is trained as described above, and the final output is a fitted neural network model (a set of network weights).

### netSNP Validation Test Using BD Populations

We conducted simulations to rule out the possibility that the netSNP method may choose SNPs based on genetic linkage to APOE ε2 or ε4; i.e., significant tSNPs could display at-risk or protective properties despite their not being pathophysiologically associated with AD. Furthermore, other details of the netSNP method may predispose cases to artifactual correlations with age of AD diagnosis and Braak scores (We note, however, that neither the age of AD diagnosis, nor their Braak score, was used in any calculations performed in section “NN Construction and Performance” or “netSNP Description and Application”).

We thus tested for the correlations shown in section “NN and CV as Predictors of AD and Its Pathophysiology.” for BDs 1–12 (see above; Supplementary Table 3 and Supplementary Figure 7). Age of diagnosis of BD was ascribed based on APOE SNPs effects in age of AD diagnosis (using MATLAB empirical cumulative distribution functions). For each BD, a balanced dataset was constructed (as for AD, see section “Dataset Pipeline, Case:Control Balancing and SNP Properties”), and BD “protective” and “at-risk” ***tSNP***s were identified as described for AD in section “NN and CV as Predictors of AD and Its Pathophysiology.” Next, we considered the set of individuals ascribed BD. We computed a correlation probability, based on a general linear model, between their age of BD diagnosis and the number of BD ***tSNP***s or number of BD CVt-weighted ***tSNP***s. Results for one BD (based on a BD constructed from BDgene CHSY1; Supplementary Figure 7D) is compared with results for AD (Supplementary Figure 7C). A summary of results for the 12 separate BDs, and AD for comparison, are shown in Supplementary Table 3. Note that for no BD was there a significant correlation (right columns). These simulations support the view that the netSNP method is not choosing AD “protective” and AD “at-risk” SNPs based on genetic linkage or some other bias introduced in the netSNP procedure.

### Statistics

Statistical methods described per figure below.

For each BD constructed, individuals in the ADSP population were assigned a BD based on their genotype; those with *APOE*_ε_2-like SNPs were randomly assigned as control with OR 2.41; those with *APOE*_ε_4-like SNPs were assigned as case with OR 0.30. Those without either SNPs were assigned randomly to control with OR 0.89 (see Supplementary Table 3). To generate random Q-Q plots, 100 datasets were generated with randomly scrambled case-control labels. Fisher’s exact test *p*-values were then computed for those 100 scrambled sets. Scrambled sets were plotted against each other to generate the C.I. region (gray dots) and also plotted against the actual data (colored dots).

Hundred random groups were generated with cases and controls counterbalanced within cohorts to formulate neural network training matrices. As described above, in each run one of these random groups was selected and an artificial NN was trained using the 50 SNPs with the lowest Fisher’s exact test *p*-value among training group individuals. NN classifier performance on the holdout set was then evaluated. A histogram of each individual’s mean NN classifier value (CV). Shows receiver operator characteristic (ROC) curves using SNP sets as features and normalizing CVs to range between 0 and 1: curve “1–50” used 50 SNPs with the lowest training group *p*-values; “1–50 *-APOE*” used 50 SNPs with the lowest training group *p*-values omitting *APOE* and *TOMM40*; “1–50 -GWAS” used 50 SNPs with the lowest training group *p*-values omitting SNPs that previously met genome-wide significance in the literature; “51–100” used SNPs with the 51st–100th lowest training group *p*-values. The left panel shows the mean correct predictions in percent for each condition in (Figure 2B); the right panel was generated like the left panel’s “Top 50,” except the experimental manipulation varied the number of samples in the training group (1 sample = 1 chromosome), as indicated in the figure legend. CVs were generated like in “2B 1–50,” and normalized to a range between –0.5 and 0.5. The classification threshold was fixed at 0 and the false discovery rate (FDR) and positive predictive value (PPV) were then computed at each corresponding *x*-axis case prevalence. The FDR and PPV were also computed using the optimal operating point (OOP):

$$S=\frac{\text{Cost}\,\left(P|N\right)-\text{Cost}\,\left(N|N\right)}{\text{Cost}\,\left(N|P\right)-\text{Cost}\,\left(P|P\right)}*\frac{N}{P}$$

where Cost(*N*| *P*) is the cost of misclassifying a case, Cost(*P*| *N*) is the cost of misclassifying a control, where *P* = *TP* + *FN*, and *N* = *TN* + *FP* (*TP*, true positive; *TN*, true negative; *FP*, false positive; *FN*, false negative). The *OOP* was then determined by moving a line with slope *S* from *FPR* = 0, *TPR* = 1 (the top left of the ROC) down-and-right, until it intersected with the ROC curve (Mathworks, 2020b).

The histograms shown in (Figure 3A) are the result of training an NN using individuals of all *APOE* subtypes, and applying this NN on holdout set individuals assigned to each of the six *APOE* genotypes. That is, after the NN is trained as described above in *General Data Preprocessing*, all holdout individuals are assigned the *APOE*ε22 genotype and a histogram is generated; then all holdout individuals are assigned the *APOE*ε*23* genotype and another histogram is generated, etc. We call this genotype assignment procedure the *netSNP method* (described below) which we show can be used as a general method for assessing the importance of any SNP on NN performance. For comparison, histograms shown in (Figure 3B) are the result of training an NN using a balanced set of individuals, and computing CVs for holdout set subgroups of individuals with the *APOE* genotypes limited to one of *APOE*ε22, ε23, ε24, ε33, ε34, or ε44. **netSNP method:** 4,000 target SNPs were chosen based on them having the lowest Fisher’s exact test *p*-value. For each target SNP, the netSNP method can produce a *NAT*, *REF*, *ALT*, and *DIF* value for each individual. For a single target SNP, obtaining these values involved the following steps: (1) a target SNP was selected to be part of a 50-SNP training matrix. (2) A random subset (∼70%) of a balanced set of individuals served as a training set. (3) From this training set, a Fisher’s exact test *p*-value was calculated for each of the (∼1.4 million) SNPs. (4) A single target SNP was paired with the 49 SNPs with the lowest *p*-value to generate a neural network training matrix. (5) The neural network was trained as described above in the *General Data Processing* methods. (6) A CV score was generated for each of the individuals in the holdout set (*NAT* score). (7) All holdout individuals were assigned the homozygous reference genotype for the target SNP and again a CV was generated (*REF* score). (8) All holdout individuals were assigned the homozygous alternate allele (minor frequency allele) for the target SNP and a CV was generated (*ALT* score). (9) The difference between the *ALT* and *REF* scores were computed (*DIF* score). This procedure was performed 20x for each target SNP; for a given target SNP, each individual’s average *ALT* score represents that individual’s ***CVt*** score for the given target SNP. In this study we tested if ***CVt*** value could be considered a weighted measure of the impact of target SNP ***t*** on the NN. Similar to how histograms are generated for, after the NN was trained as described above in *General Data Preprocessing*, all holdout individuals were assigned the homozygous genotype for minor frequency allele of the target SNP for the indicated gene (see Table 1 for chromosome and position of the target SNP for each indicated gene).

Boxplots in (Figure 4A) were generated by grouping case individuals based on whether they had a homozygous reference, heterozygous, or homozygous minor frequency for the indicated allele, and plotted the median AD age-of-onset (+/– interquartile range, IQR; whiskers = range; dots = outliers). Boxplots were generated by pooling case individuals into six bins that were uniformly discretized based on the NN CV value, on the number of protective (left) or risk (right) target SNPs each individual had, or ***CVt***-weighted target SNPs, and then plotted the median AD age-of-onset (+/– IQR; whiskers = range; dots = outliers) for each of these bins. Figure 4E (left) was generated like Figure 4C, considering previously published (without *APOE*) AD SNPs. Figure 4D (right) was generated like Figure 4C, providing a netSNP-computed ***CVt*** for each previously published (without *APOE*) AD SNPs. Brain sections in (Figure 4F) depict Braak staging – a method used to classify the degree of pathology in Alzheimer’s disease – commonly used in post-mortem clinical diagnosis of AD by performing brain autopsy; images here intend to summarize the general disease sequelae as shown in actual brain images from Braak et al. (2006). The bar plot in (Figure 4G) was generated by identifying individuals that had ***mCVt*** scores across all ***tSNP***s that fall into each of the indicated bins, and the mean Braak stage of the individuals in each bin was plotted. Boxplots in (Figure 4H) pool individuals based on ADSP-reported Braak values and plot the median number of target SNPs (+/– IQR; whiskers = range; dots = outliers) found in individuals with a brain pathology that fall into one of these six Braak stages; as in (Figures 4C,D), effects are shown separately for SNPs that potentially confer protection (left panel) and risk (right panel). *P*-values were computed using a general linear model, where *p*-value represents the probability of the slope coefficient having such a magnitude, under the null hypothesis.

## Data Availability Statement

The dataanalyzed in this study is subject to the following licenses/restrictions: NIH ADSP Embargo – Access granted via application. Requests to access these datasets should be directed to https://dss.niagads.org/documentation/applying-for-data/application-instructions/.

## Ethics Statement

Ethical review and approval was not required for the study on human participants in accordance with the local legislation and institutional requirements. The patients/participants provided their written informed consent to participate in this study.

## Author Contributions

BM and RM designed the study. BM, SP, TG, and RM prepared and preprocessed the data. BM, AnR, AlR, and RM performed the statistical analyses. BM and RM generated figures. BM, AlR, and RM wrote the manuscript. All authors contributed to the article and approved the submitted version.

## Conflict of Interest

The authors declare that the research was conducted in the absence of any commercial or financial relationships that could be construed as a potential conflict of interest.

## Supplementary Material

Supplementary Figure 1 A multi-layer feed forward neural network was trained to classify individuals by cohort identity. The scaled conjugate gradient (SCG) algorithm was the primary learning algorithm used to minimize neural network weights, here and in all other applications of a neural network in this manuscript unless otherwise noted.

Supplementary Figure 2 ROC curves of NN performance were generated based on neural net output after being trained on individual cohort labels, and using individual genotypes (for the top 50 SNPs) as training features.

Supplementary Figure 3 For neural net architecture used to classify individuals as cases or controls see methods for Supplementary Figure 1.

Supplementary Figure 4 See legend.

Supplementary Figure 5 Distributions of ***CVt*** values are based on netSNP outputs (see **netSNP method** above). To determine if any ***tSNPs*** might confer protection from *APOE*ε4 effects, neural nets weights were fit using true training group genotypes; then the netSNP test was performed simultaneously for *APOE*ε4 and the ***tSNP***, such that in each holdout group individual the *APOE* locus was set to ε4 and the target SNP locus were set to the homozygous minor alleles, and neural net output (***CVt***) was evaluated.

Supplementary Figure 6 The ***CVt*** distributions of holdout group individuals for different ***tSNP***s were generated using the netSNP method (see **netSNP method** above); here *APOE* and *TOMM40* were excluded as training features.

Supplementary Figure 7 Same methods used to generate Figure 4, with the only modification being that *APOE* and *TOMM40* were excluded as training features, except Panel-A which necessarily includes *APOE* as a training feature. The left boxplot of Panel-E is the same as in Figure 4 since the raw per-person count of known AD genes is independent of neural network output and netSNP manipulations.

Supplementary Figure 8 See text: results section “netSNP as Predictor of AD-Linked *tSNPs* and AD Pathophysiology.” For cases, ages of BD diagnosis was assigned based on AD age distribution for analogous genotype [e.g., those with SNP2 (E4-like) were given age of diagnosis with a distribution as individuals with *APOE*ε4 are diagnosed with AD]. For each BD, netSNP analysis was performed as it was for AD (**netSNP method**, above). Correlations between age of BD diagnosis and 12 of netSNP-identified SNPs and CVt-weighted 12 of netSNP-identified SNPs was conducted as for AD (Figure 4).

Supplementary Figure 9 Same methods used to generate Figure 2D using a classification threshold fixed at zero. NCASE, the number of individuals in the case condition; PCASE, the number of individuals predicted as case; NCTRL, the number of individuals in the control condition; PCTRL, the number of individuals predicted as control; TP, true positive; TN, true negative; FP, false positive; FN, false negative; TPR, true positive rate; TNR, true negative rate; FPR, false positive rate; FNR, false negative rate; PPV, positive predictive value; NPV, negative predictive value; FOR, false omission rate; FDR, false discovery rate; AUC, area under the curve; ACC, accuracy; FOS, F1-score; PLR, positive likelihood ratio; NLR, negative likelihood ratio; DOR, diagnostic odds ratio; MCC, Matthews correlation coefficient.

Supplementary Figure 10 Same methods used to generate Figure 2D using the OOP as the classification threshold. Same abbreviations as in Supplementary Figure 9.

Supplementary Figure 11 Definitions in Supplementary Figure 9 shown in the form of a confusion matrix.

Supplementary Figure 12 See Figure 1C.

Supplementary Figure 13 See Figure legend.
