# Supplementary material for: A Machine Learning Method to Identify Genetic Variants Potentially Associated With Alzheimer’s Disease
Source: Front Genet. 2021 Jun 14;12:647436. doi: 10.3389/fgene.2021.647436 (PMC8238203; doi:10.3389/fgene.2021.647436)
Supplement: Supplementary Figure 1 — A multi-layer feed forward neural network was trained to classify individuals by cohort identity. The scaled conjugate gradient (SCG) algorithm was the primary learning algorithm used to minimize neural network weights, here and in all other applications of a neural network in this manuscript unless otherwise noted. [file Data_Sheet_1.PDF]

Figures & Tables

Figure 1 .... pp. 2
Figure 2 .... pp. 3
Figure 3 .... pp. 4
Figure 4 .... pp. 5
Table 1 .... pp. 6

Figure S1 .... pp. 8
Figure S2 .... pp. 9
Figure S3 .... pp. 10
Figure S4 .... pp. 11
Figure S5 .... pp. 12
Figure S6 .... pp. 13
Figure S7 .... pp. 14
Figure S8 .... pp. 15
Figure S9 .... pp. 16
Figure S10 .... pp. 17
Figure S11 .... pp. 18
Figure S12 .... pp. 19
Figure S13 .... pp. 20

Table S1 .... pp. 21
Table S2 .... pp. 22
Table S3 .... pp. 23

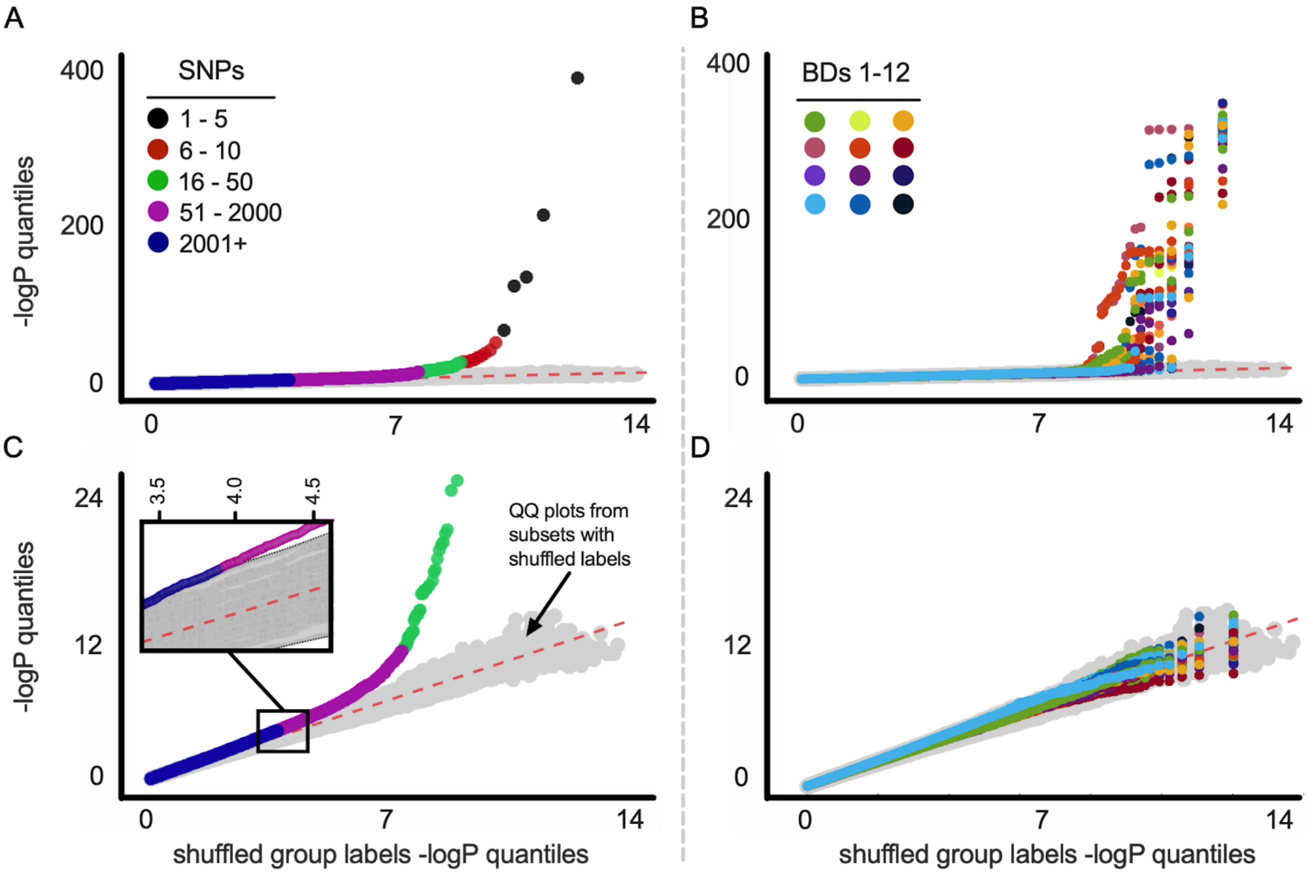

Figure 1. ADSP data processing and SNP frequency distribution.

A. Q-Q plots of balanced ADSP dataset for Alzheimer's Disease. Gray symbols (here, and below) represent Q-Q plot of 100 random vs random distributions (i.e. chance). See text for details. SNP order (based on -log(P)) is indicated by colors (see legend).

B. Q-Q plots of balanced ADSP dataset for 12 constructed (simulated) diseases (BDs). Each BD is represented by a different color.

C. Same as above with SNPs from APOE-residing chromosome 19 removed before p-value quantiles were computed. Inset is a blow up of indicated region, showing magenta SNPs fall outside the 100 random vs random distributions (gray symbols). SNP order (based on -log(P)) is indicated by colors (see legend).

D. Same as above with SNPs from BDgene-residing chromosome removed before p-value quantiles were computed. Plot as in panel B is shown for all BD1-33 in Figure S12.

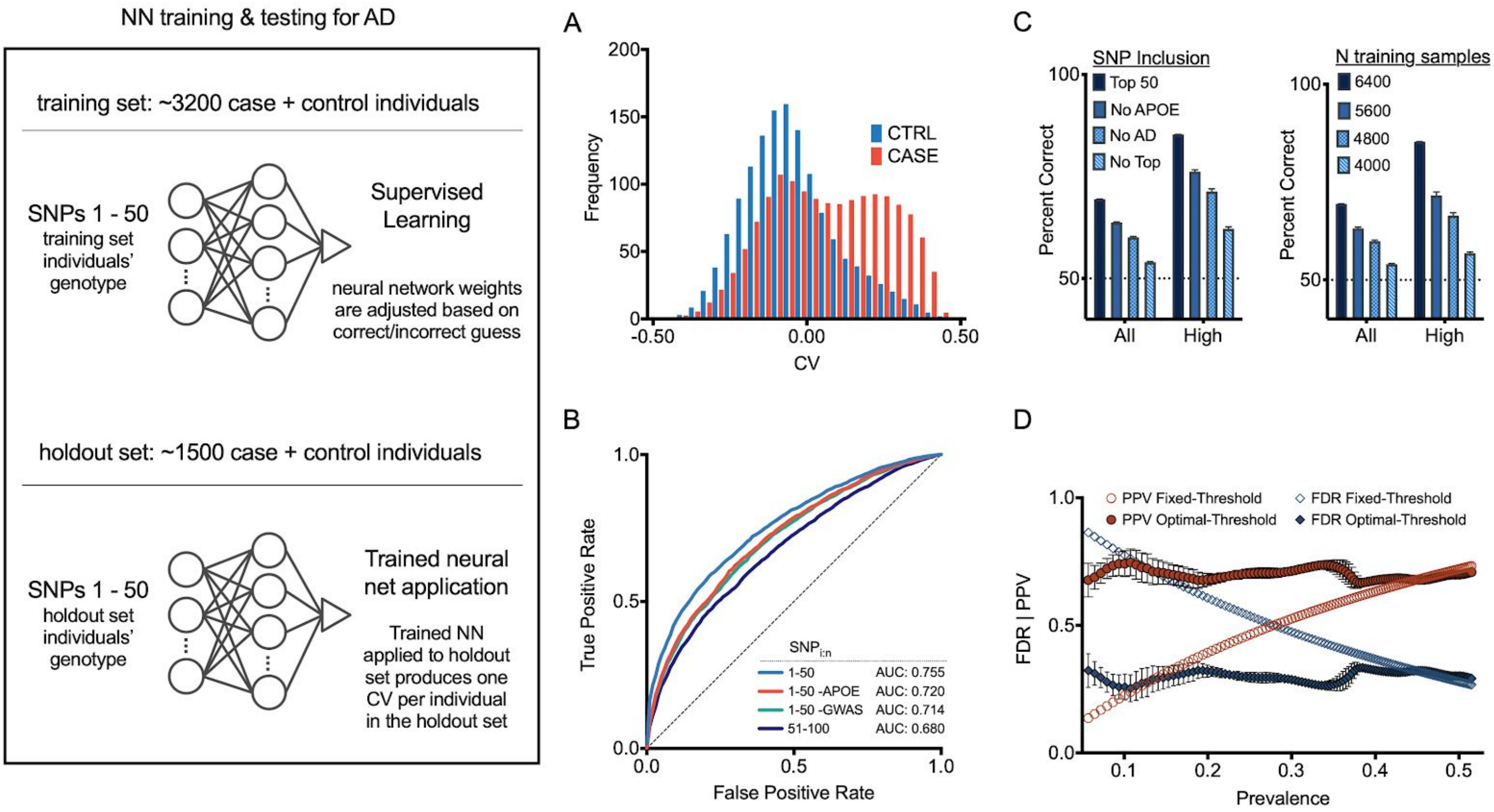

Figure 2. Neural net prediction of case and control individuals

Left Panel: general neural net protocol and architecture; see text for details.

A. Histogram of neural net confidence value (CV) output for case (red) and control (blue) holdout set individuals.

B. Receiver operator characteristic (ROC) curves for indicated SNP sets as features.

C. (Left) Neural net accuracy for indicated populations using indicated SNP sets as features. 'All CV' and 'High abs(CV)' indicates inclusion of populations with 100% or top 30% abs(CV) values in accuracy calculations. Error bars, SEM. (Right) Neural net accuracy with indicated size training set; two samples (chromosomes) per individual.

D. False discovery rate (FDR) and Positive Predictive Value (PPV) at each corresponding x-axis case prevalence, using a fixed CV threshold (set to 0) or optimal operating point classification threshold (see *Methods*). Additional measures in Figures S9-S11.

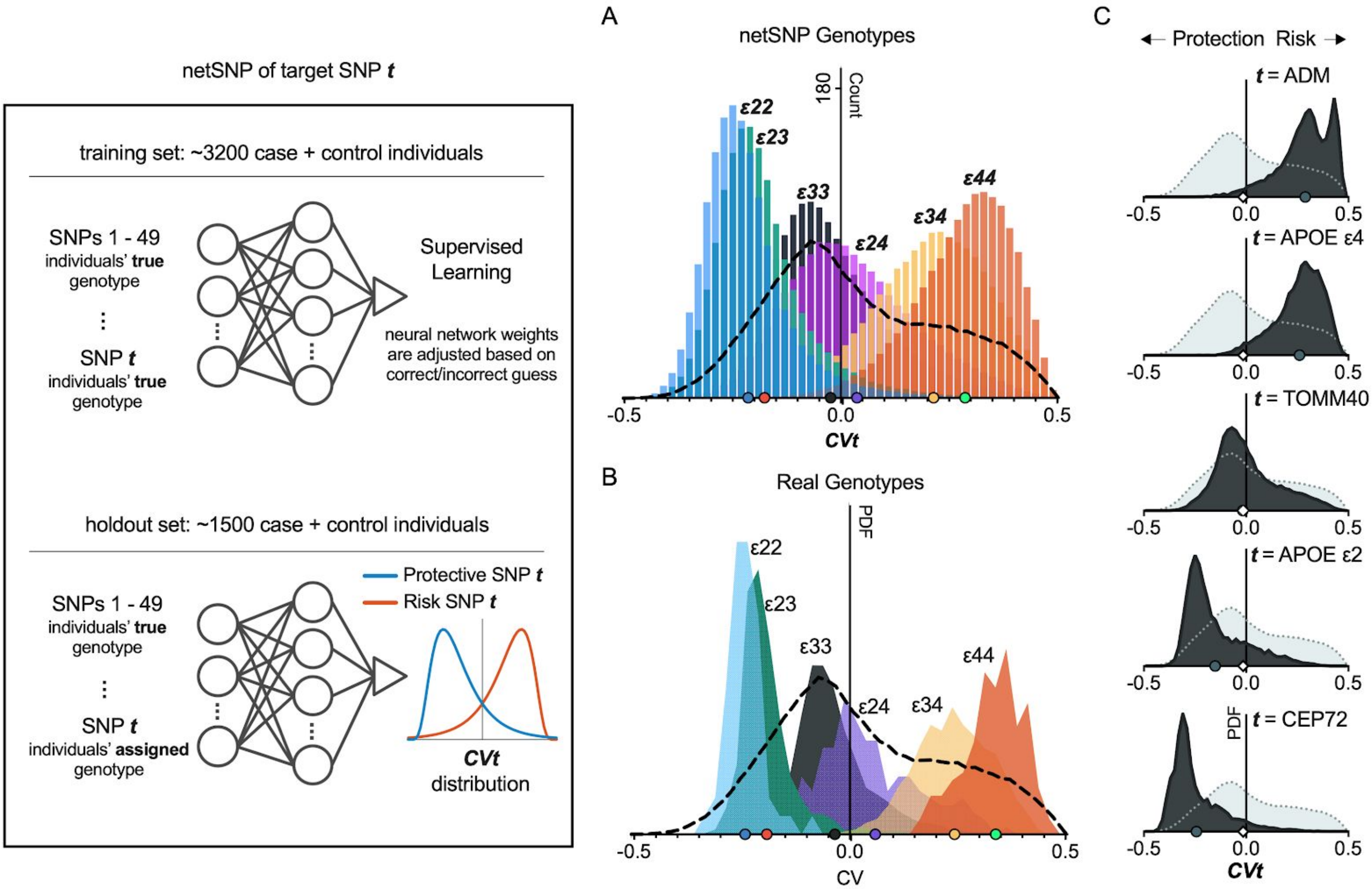

Figure 3. netSNP accurately reproduces CV values for APOE genotypes and identifies potential AD-risk and AD-protective SNPs

Left Panel: Diagram of netSNP method; details in text. SNP ***t*** assignment, APOE genotype for A; and homozygous MFA for C.

A. netSNP-generated CVt values for all holdout set individuals (all APOE genotypes) with their genotype artificially assigned to indicated genotype. Dashed line indicates distribution of CV values for all holdout set individuals with correct genotype.

B. Frequency distribution of CV values for holdout set subsets containing only individuals with indicated APOE genotype. Dashed line as above.

C. Example netSNP-generated CV distributions for all holdout individuals with true genotype (light gray) or CVt with indicated target SNPs (dark gray) assigned alt/alt; symbols on X axis: mean CVt (mCVt) values. Note that mCVt value for TOMM40 SNP is close to zero, indicating that it perturbs NN output little (i.e. provides little additional information) when APOE SNPs are used in training. ***CVt*** distributions for fifty ***tSNPs*** shifting CV most to the left and fifty ***tSNPs*** shifting CV most to the right are shown in Figure S5. ***tSNPs*** having potentially a protective effect on individuals with *APOE4* is shown in Figure S5; Table S2 and online.

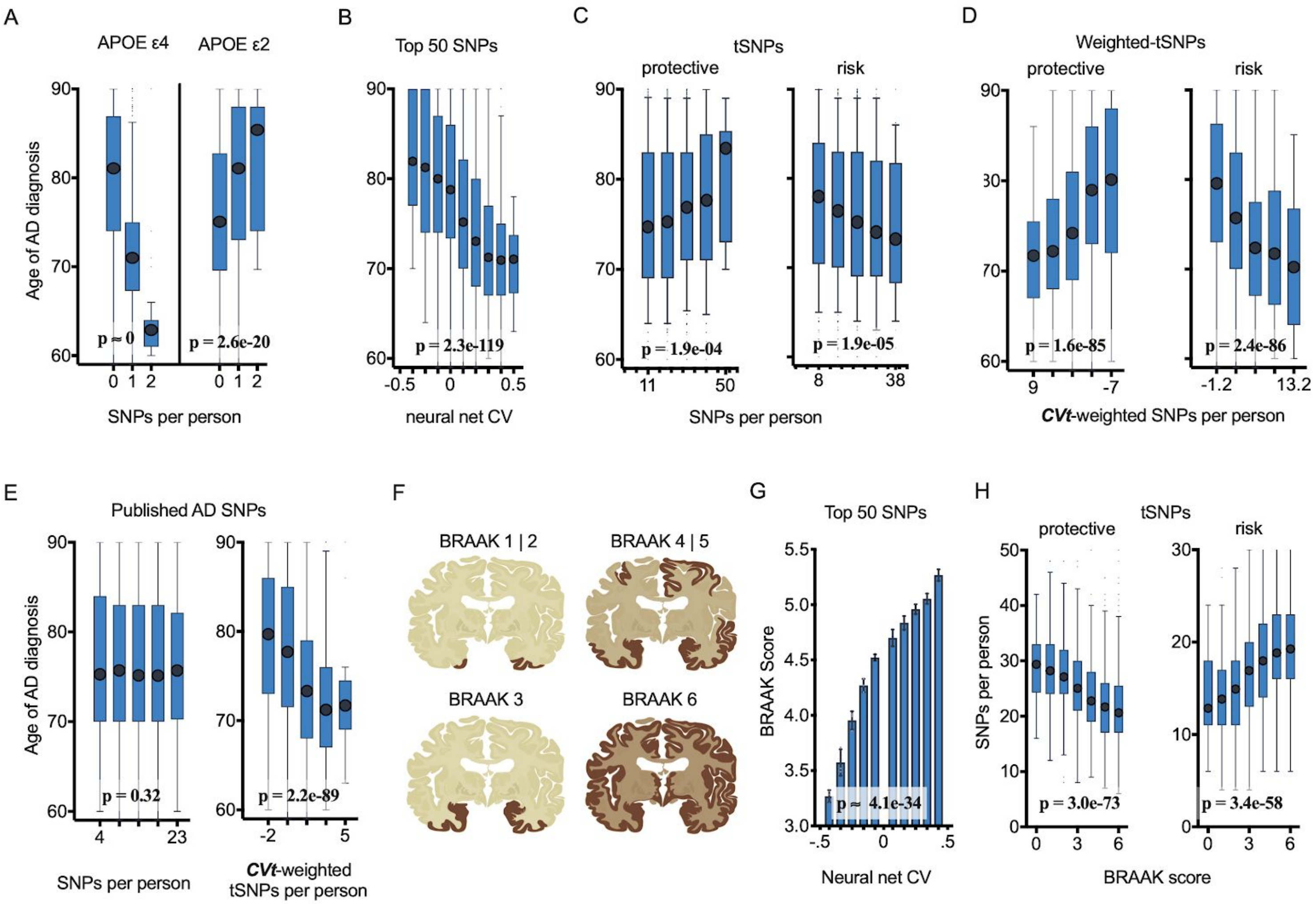

Figure 4: netSNP validation: Number of netSNP-identified tSNPs and netSNP-CVt-weighted tSNPs correlate with age of AD diagnosis of all case individuals (N=4752) and AD pathology (case and control receiving autopsy, N=2700). (See supplementary Figure S8 for results excluding APOE and TOMM40 in netSNP training matrices; results are similar; conclusions are the same).

A. Age of AD diagnosis plotted against number of APOE e4 (or APOE e2) SNPs per person. Here and below: boxplot X-axis values indicate mean value of boxed group; p values based on general linear model analysis of variance.
B. Age of AD diagnosis plotted versus 50 SNP neural net CV.
C. Age of AD diagnosis plotted versus number of netSNP-identified AD-protective, left, or AD-risk tSNPs.
D. Age of AD diagnosis plotted versus netSNP-CVt-weighted number of AD-protective (left) and AD-risk (right) tSNPs.
E. Age of AD diagnosis plotted versus number (left) or netSNP-CVt-weighted number (right) of previously published AD-linked SNPs, excluding those in APOE/TOMM40. n.s., not significant.
F. Cartoon diagram of human brain with affected regions for indicated Braak scores.
G. Neural net CV plotted versus Braak score.
H. netSNP-identified AD-protective (left) and AD-risk (right) tSNPs per person plotted versus Braak score.

Table 1. netSNP identified tSNPs with greatest absolute average ***CVt*** when APOE locus variants were not excluded from the training set

tSNPs predicted to confer most protection against AD

Chr	Pos	Gene	***mCVt***	FishP	MAF
5	612,536	CEP72	**-0.243**	5.7E-03	0.002
6	1,390,303	FOXF2	**-0.182**	1.4E-02	0.003
4	110,638,764	PLA2G12A	**-0.178**	2.8E-02	0.002
1	16,890,642	NBPF1	**-0.174**	3.3E-02	0.002
11	1,017,294	MUC6	**-0.157**	1.4E-07	0.01
1	40,961,395	ZFP69	**-0.156**	4.8E-04	0.002
15	50,154,563	ATP8B4	**-0.156**	2.3E-02	0.004
19	52,793,834	ZNF766	**-0.155**	2.8E-02	0.002
15	64,017,685	HERC1	**-0.152**	3.5E-03	0.004
19	45,412,079	APOE ε2*	**-0.152**	7.1E-38	0.079
16	8,740,006	METTL22	**-0.15**	2.1E-03	0.002
9	139,396,933	NOTCH1	**-0.143**	3.3E-02	0.004
19	18,561,473	ELL	**-0.137**	7.3E-03	0.008
11	57,467,411	ZDHHC5	**-0.133**	3.5E-03	0.002
9	100,372,648	TSTD2	**-0.131**	2.2E-02	0.003
1	65,120,426	CACHD1	**-0.131**	1.0E-02	0.002
12	69,113,184	NUP107	**-0.126**	5.6E-03	0.006
5	145,508,644	LARS	**-0.126**	1.2E-02	0.006
7	6,561,105	GRID2IP	**-0.125**	2.9E-04	0.002
19	43,268,061	PSG8	**-0.125**	2.9E-02	0.004
11	47,264,353	ACP2	**-0.125**	1.4E-03	0.004
6	7,405,242	RIOK1	**-0.124**	1.5E-02	0.003
3	146,167,089	PLSCR2	**-0.123**	2.3E-02	0.003
16	30,775,522	RNF40	**-0.123**	2.9E-02	0.006
9	139,008,644	C9orf69	**-0.121**	2.9E-03	0.003

Rows 26:1000 available online

* Previously published AD-linked gene

tSNPs predicted to confer the most risk for AD

Chr	Pos	Gene	***mCVt***	FishP	MAF
11	10,327,875	ADM	**0.289**	4.1E-08	0.008
19	45,411,941	APOE ε4*	**0.261**	3.4E-111	0.135
7	23,213,734	KLHL7	**0.217**	5.1E-03	0.003
9	130,439,029	STXBP1	**0.207**	1.9E-02	0.001
20	37,258,198	ARHGAP40	**0.203**	1.1E-02	0.002
15	41,862,356	TYRO3	**0.197**	8.4E-18	0.018
6	146,276,263	SHPRH	**0.195**	6.6E-03	0.002
1	228,879,367	RHOU	**0.195**	6.4E-03	0.004
9	131,398,647	WDR34	**0.19**	7.0E-03	0.002
19	52,497,235	ZNF615	**0.188**	3.3E-02	0.003
12	85,450,243	LRRIQ1	**0.188**	1.0E-02	0.006
15	25,963,545	ATP10A	**0.185**	1.2E-02	0.002
12	108,011,971	BTBD11	**0.183**	3.8E-03	0.007
9	107,533,232	NIPSNAP3B	**0.181**	1.1E-02	0.003
1	8,420,270	RERE	**0.18**	3.0E-02	0.004
8	10,480,495	RP1L1	**0.179**	3.1E-02	0.003
4	5,682,993	EVC2	**0.178**	2.2E-02	0.004
5	140,530,973	PCDHB6	**0.178**	1.4E-02	0.002
6	30,712,298	IER3	**0.177**	3.3E-03	0.007
15	50,264,839	ATP8B4	**0.176**	1.1E-02	0.008
16	3,604,305	NLRC3	**0.176**	2.7E-02	0.002
22	46,780,446	CELSR1	**0.174**	1.9E-02	0.003
19	39,103,307	MAP4K1	**0.173**	1.9E-02	0.001
1	89,579,827	GBP2	**0.173**	2.5E-02	0.005
12	50,500,080	GPD1	**0.173**	2.6E-03	0.002

Rows 26:1000 available online

* Previously published AD-linked gene

See Table S1 for the same netSNP computations with APOE locus variants excluded from the training set; similar results hold.

Supplemental Figures & Tables

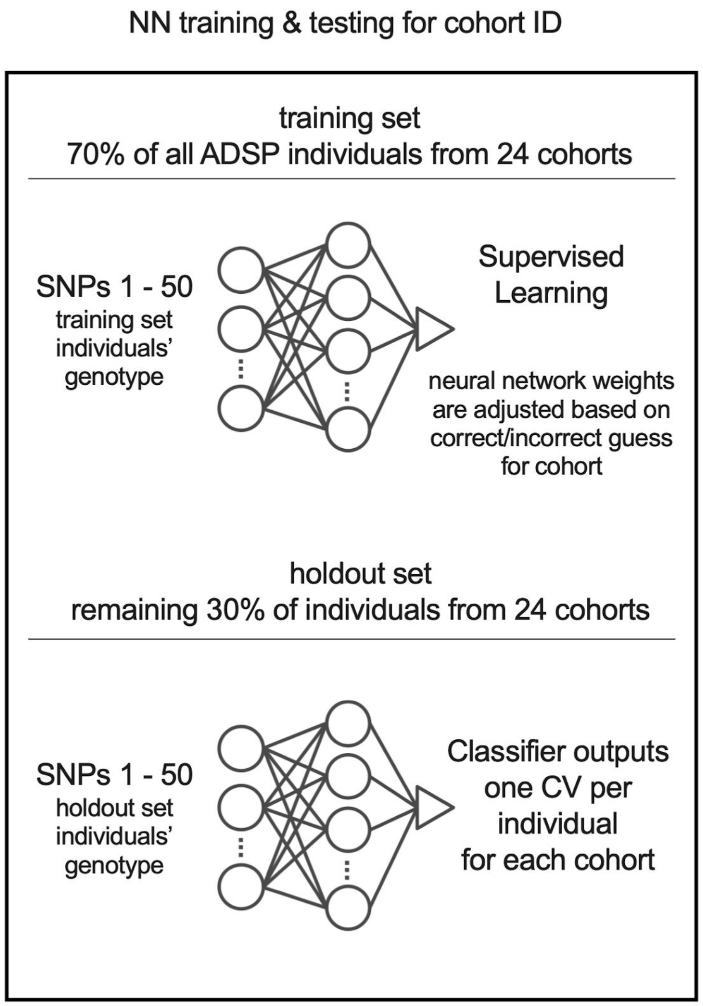

Figure S1. Neural net procedure and architecture for classifying individuals by cohort identity.

	Full Dataset		After Balancing	
	Cases	Controls	Cases	Controls
**ACT**	323	945	323	323
**ADC**	2438	817	817	817
**ARIC**	39	18	0	0
**ASPS**	121	5	0	0
**CHAP**	27	204	0	0
**CHS**	250	583	250	250
**CUHS**	160	171	0	0
**ERF**	45	0	0	0
**FHS**	157	424	157	157
**GDF**	111	96	96	96
**LOAD**	367	109	109	109
**MAP**	138	277	138	138
**MAYO**	250	99	99	99
**MIA**	186	14	0	0
**MIR**	316	15	0	0
**MPD**	0	20	0	0
**NCRD**	160	0	0	0
**RAS**	46	0	0	0
**ROS**	154	197	154	154
**RS**	276	813	276	276
**TARC**	132	12	0	0
**TOR**	9	0	0	0
**VAN**	210	26	26	26
**WCAP**	34	116	34	34

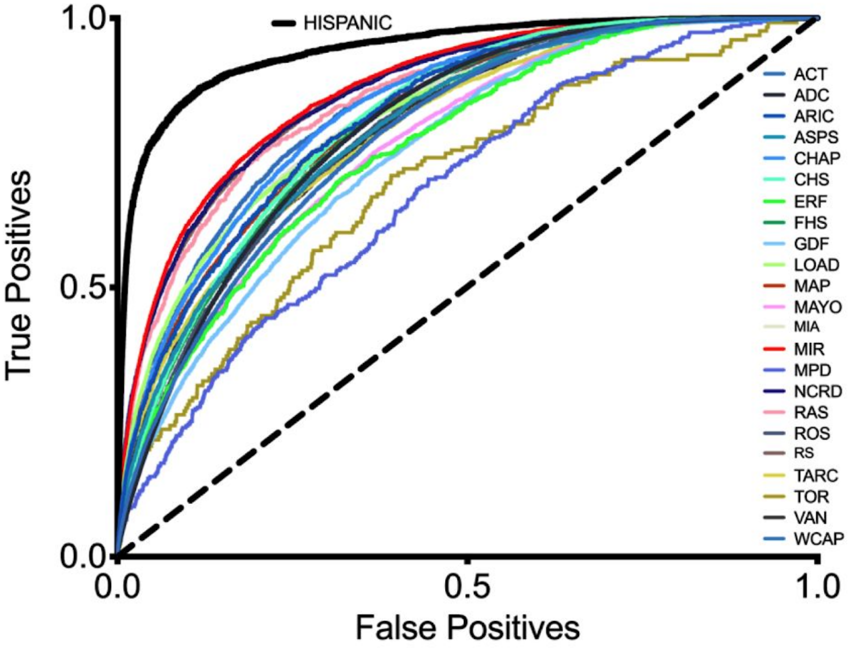

Figure S2. Left, cohort makeup of dataset before and after balancing. Cohorts with gray sections removed from analysis. Right, receiver operating characteristics (ROC) curves of NN performance after training (values for each line created by varying CV threshold); note performance is much better than chance (diagonal dashed line).

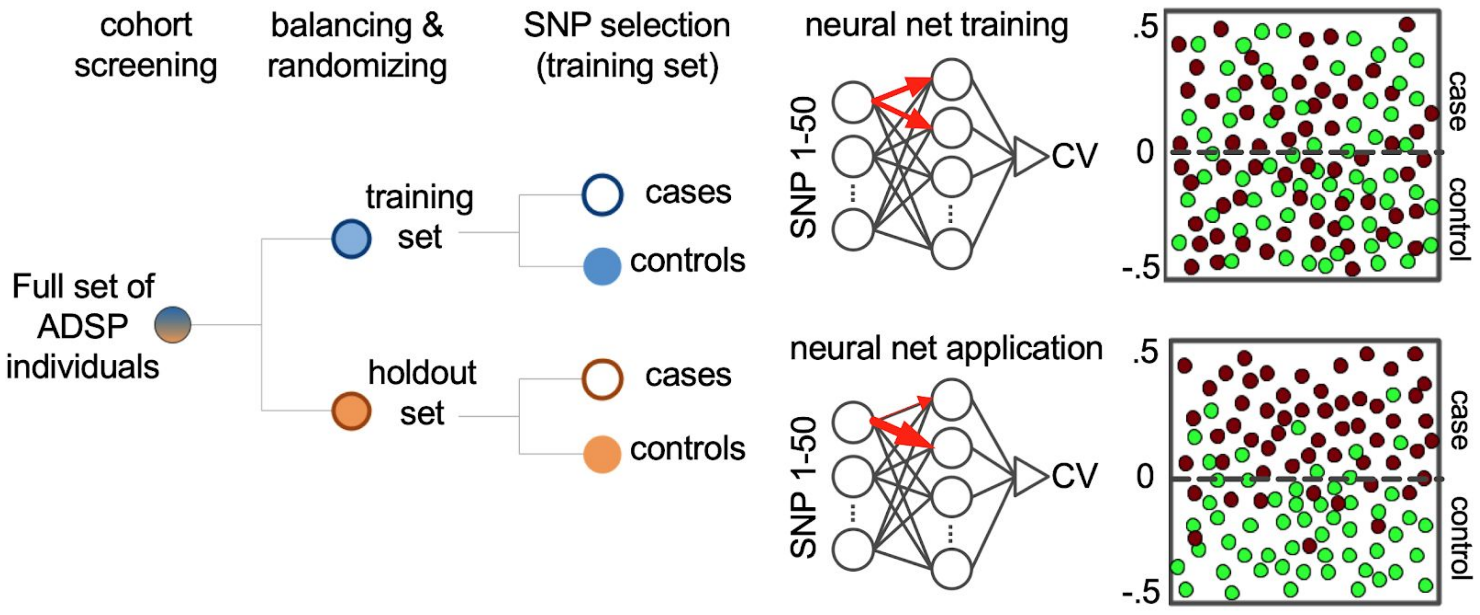

Figure S3. Neural net procedure and architecture for classifying individuals as cases or controls. Note red arrows indicate weights before (top) and after (bottom) training, for explanatory purposes.

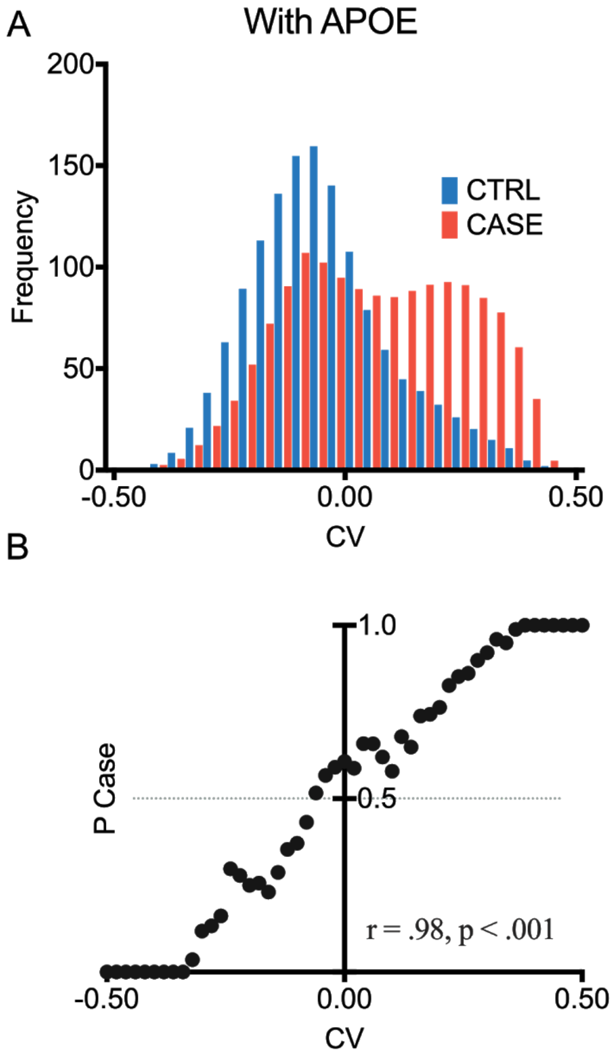

Figure S4: (A) Histogram of neural net confidence value (CV) output for case (red) and control (blue) holdout set individuals. (B) Fraction of individuals with indicated CV predicted to be a case. (C) Same as A, without APOE in CV computation. (D) Same as B, without APOE in CV computation.

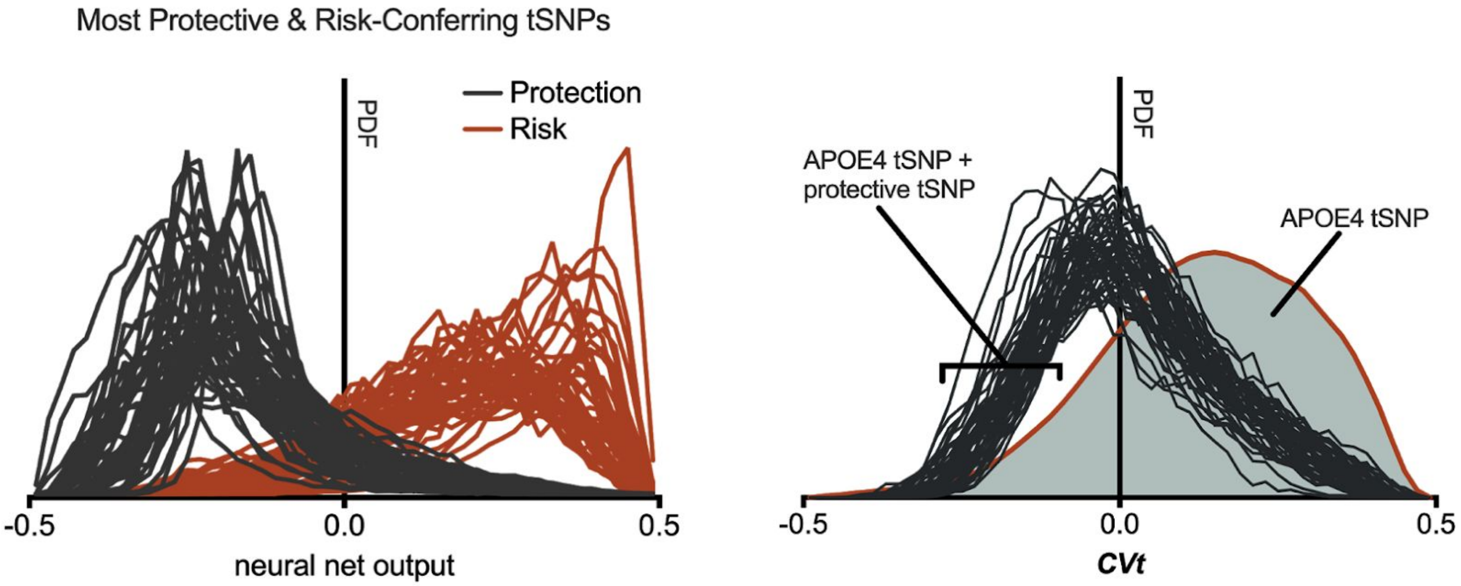

Figure S5. Left: CVt distributions for different tSNPs; right, tSNPs that confer protection for APOE4. netSNP for target SNP t, applied with holdout individuals assigned APOE34 genotype and alt/alt for SNP t. tSNP chromosome:location and CVt values given in Table S2.

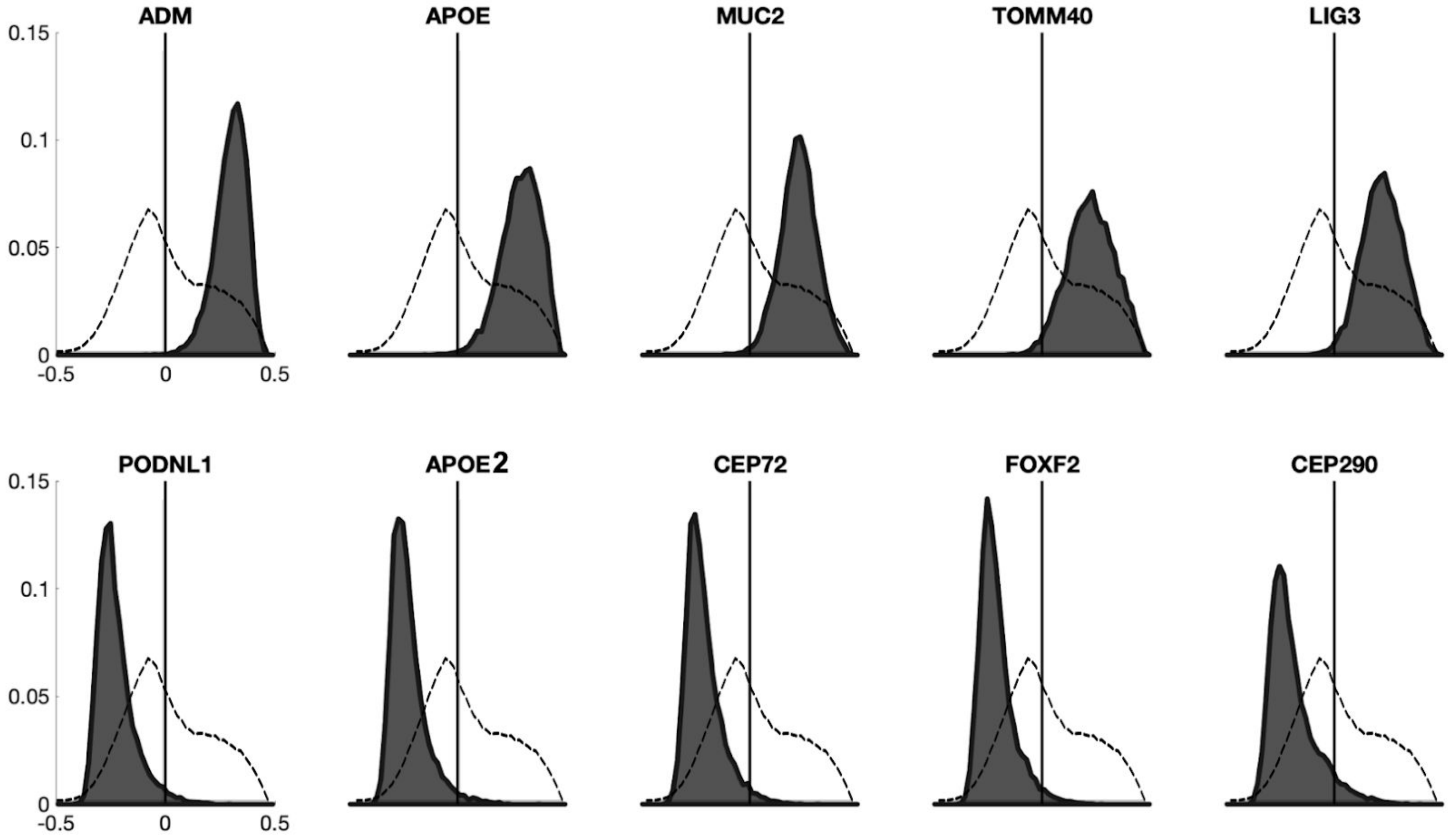

Figure S6. CVt distributions for different tSNPs, when netSNP training SNPs do not include APOE SNPs.

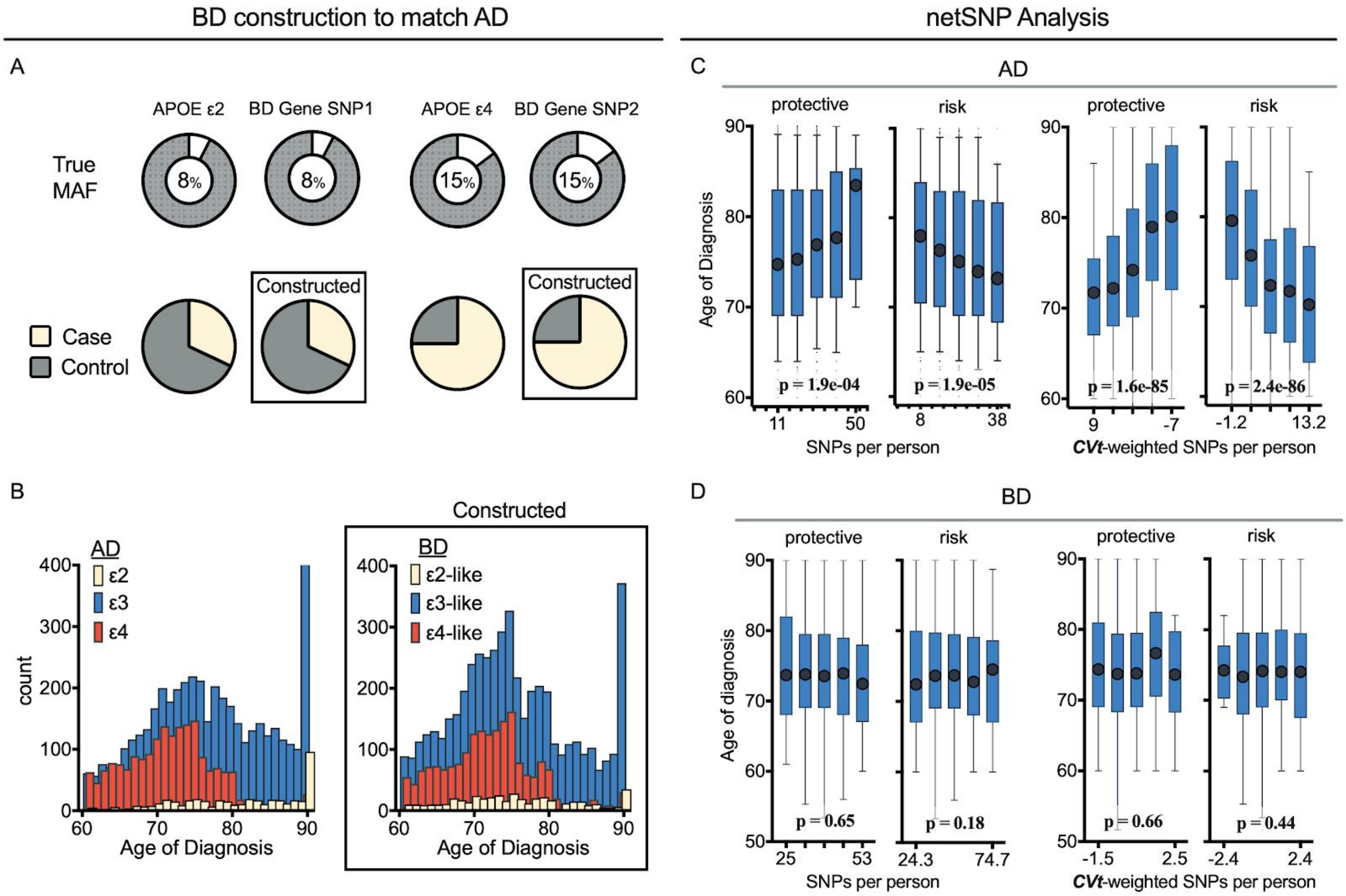

Figure S7. netSNP validation simulation: simulated diseases constructed based on APOE fail to display a significant correlation with age of disease onset.

A. 'Bad Disease (BD)' construction based on an existing gene with 2 SNPs (top) whose minor allele frequency (MAF) match APOE2 and APOE4 (pie charts; see Table 1). Bottom, case and control randomly assigned BD, constrained by BD-gene genotype; BD assignation of BD-gene genotype individuals parallels frequency of AD found in individuals with different APOE genotype.
B. Age of BD diagnosis for individuals with BD-gene genotype (right) randomly assigned as indicated in A, matches the age distribution of AD for APOE genotypes (left).
C. netSNP analysis for AD (same as Figure 4C, 4D), to be compared with D.
D. netSNP analysis for BD based on an existing gene, CHSY1. See *Methods* for details and Table S3 for summary of all BDs.

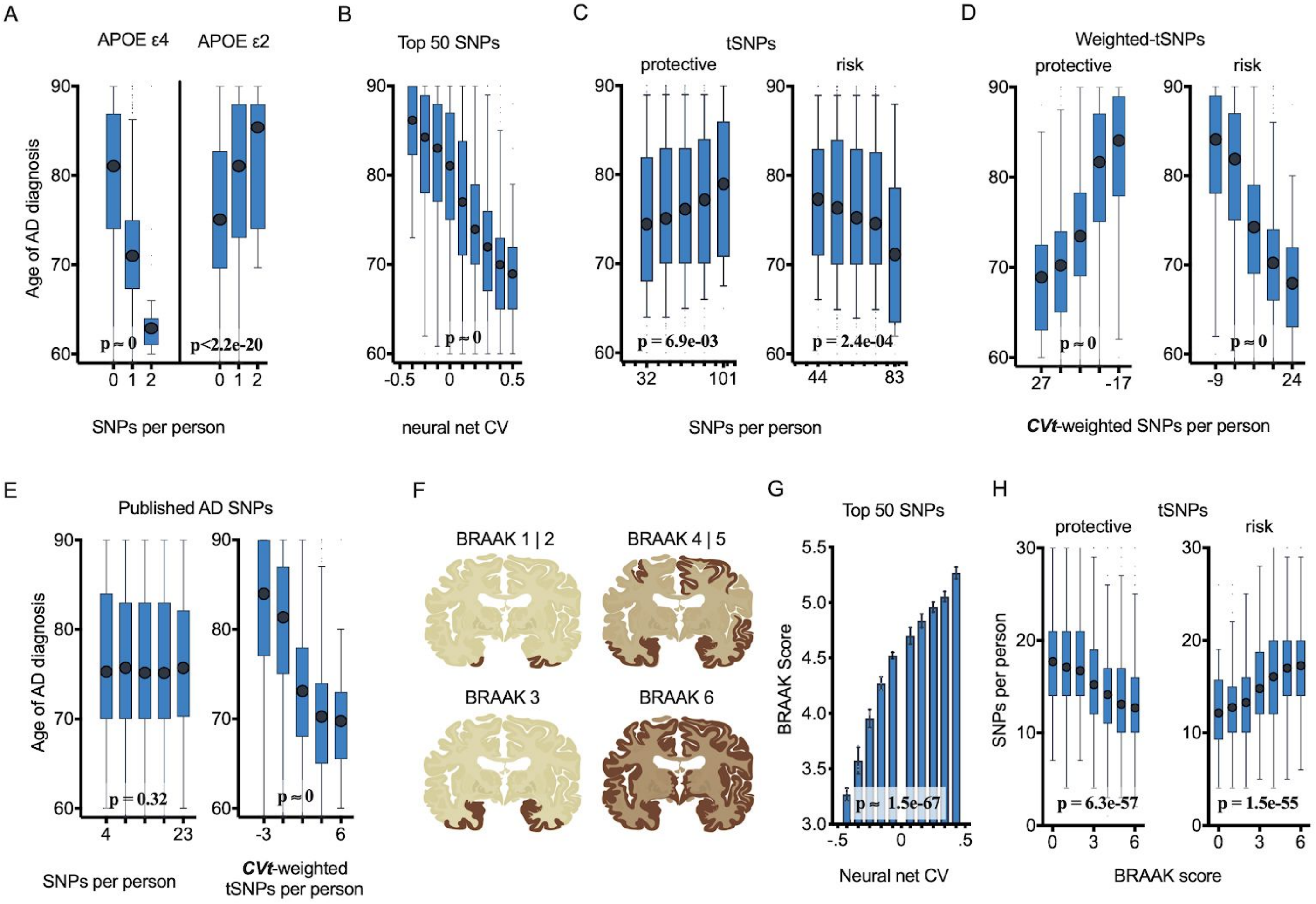

Figure S8. netSNP correlations as in main Figure 4, when netSNP training SNPs do not include APOE SNPs.

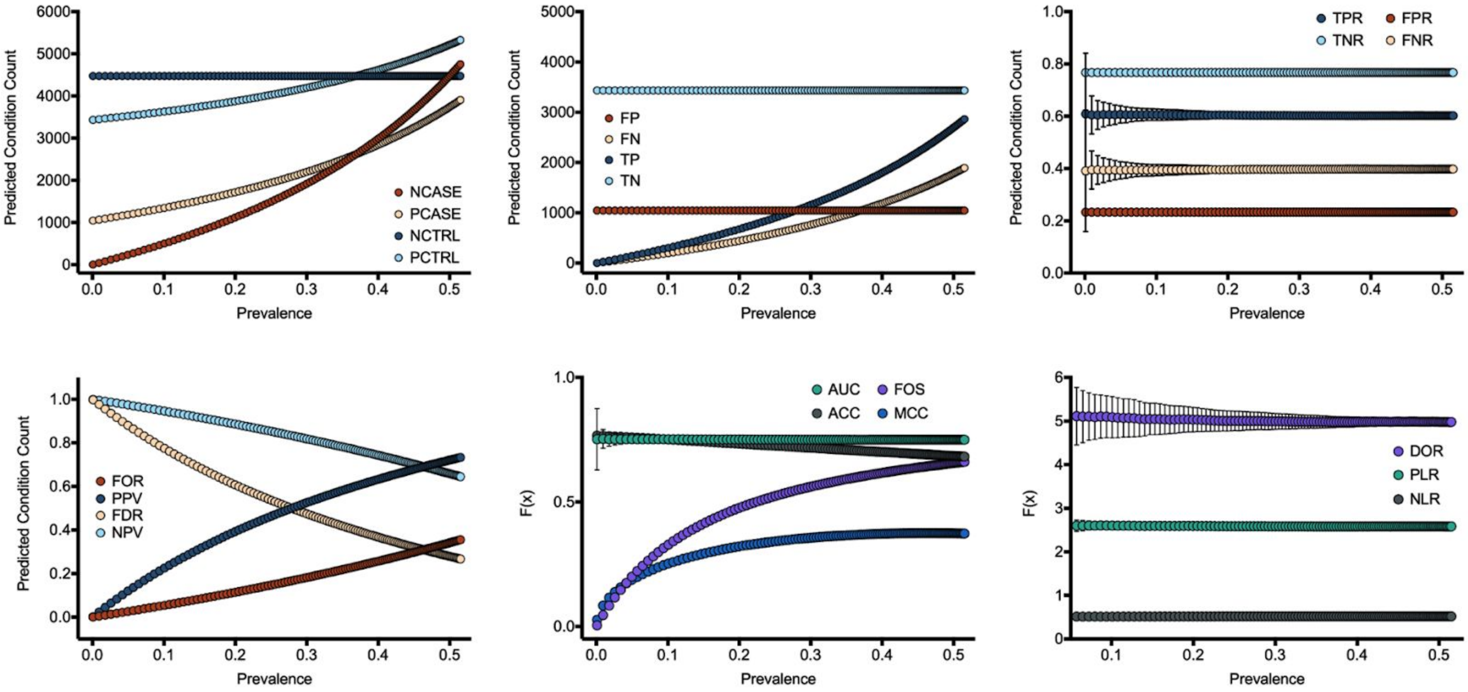

Figure S9. Confusion matrix statistics derived using a fixed operating point threshold. Abbreviations are defined in the confusion matrix shown in Figure S11.

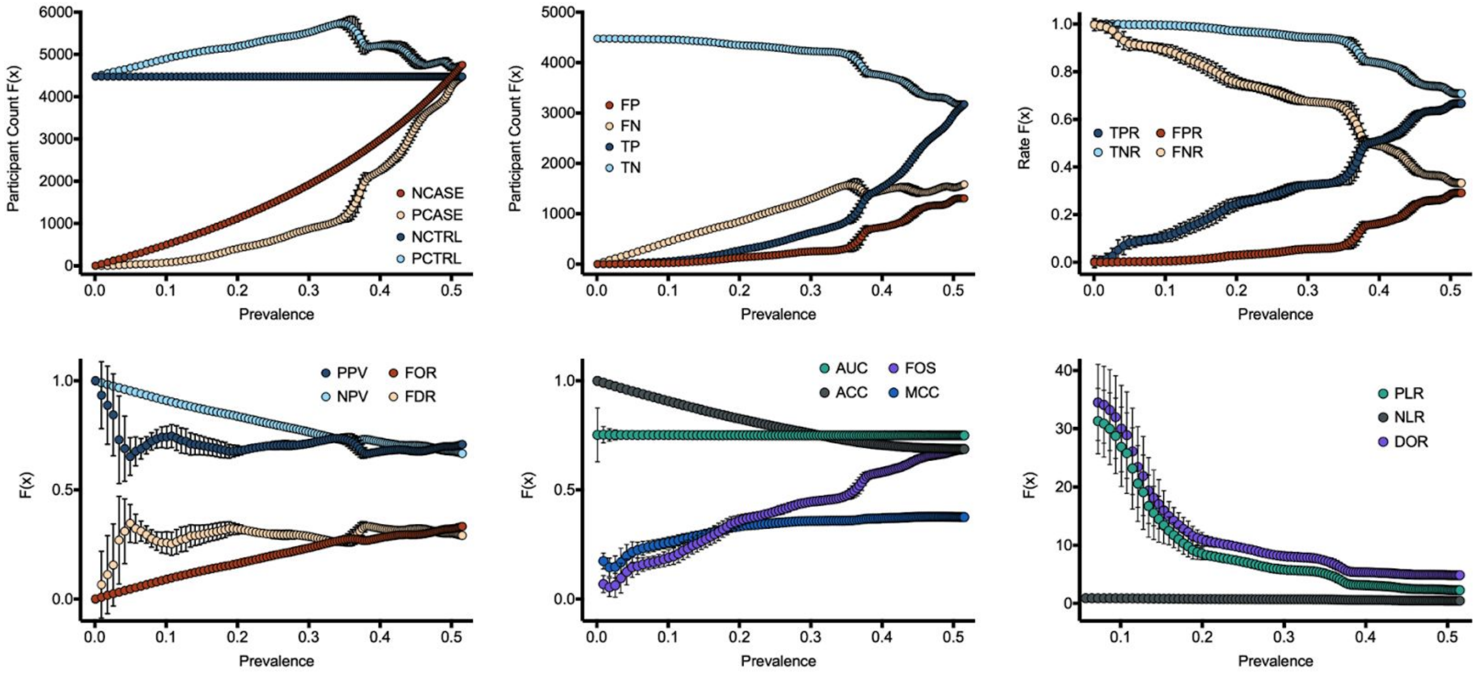

Figure S10. Confusion matrix statistics derived using the optimal operating point threshold. Abbreviations are defined in the confusion matrix shown in Figure S11

Condition

Predicted				
Total (*Pop*)ulation	$NCond(+)$	$NCond(-)$	$(Prev)alence = \frac{Cond(+)}{Pop}$	$(Acc)uracy = \frac{(TP+TN)}{Pop}$
$NPred(+)$	True Positive TP	False Positive FP	Positive Predictive Value $PPV = \frac{TP}{Pred(+)}$	False Discovery Rate $FDR = \frac{FP}{Pred(+)}$
$NPred(-)$	False Negative FN	True Negative TN	False Omission Rate $FOR = \frac{FN}{Pred(-)}$	Negative Predictive Value $NPV = \frac{TN}{Pred(-)}$
AUC	True Positive Rate $TPR = \frac{TP}{Cond(+)}$	False Positive Rate $FPR = \frac{FP}{Cond(-)}$	Positive Likelihood Ratio $PLR = \frac{TPR}{FPR}$	Diagnostic Odds Ratio $DOR = \frac{PLR}{NLR}$
OOP	False Negative Rate $FNR = \frac{FN}{Cond(+)}$	True Negative Rate $TNR = \frac{TN}{Cond(-)}$	Negative Likelihood Ratio $NLR = \frac{FNR}{TNR}$	F1-Score $FOS = 2 * \frac{PPV * TPR}{(PPV + TPR)}$

Figure S11. Confusion matrix; in Figures S9 and S10 a plot of each panel is given across a range of AD population prevalences

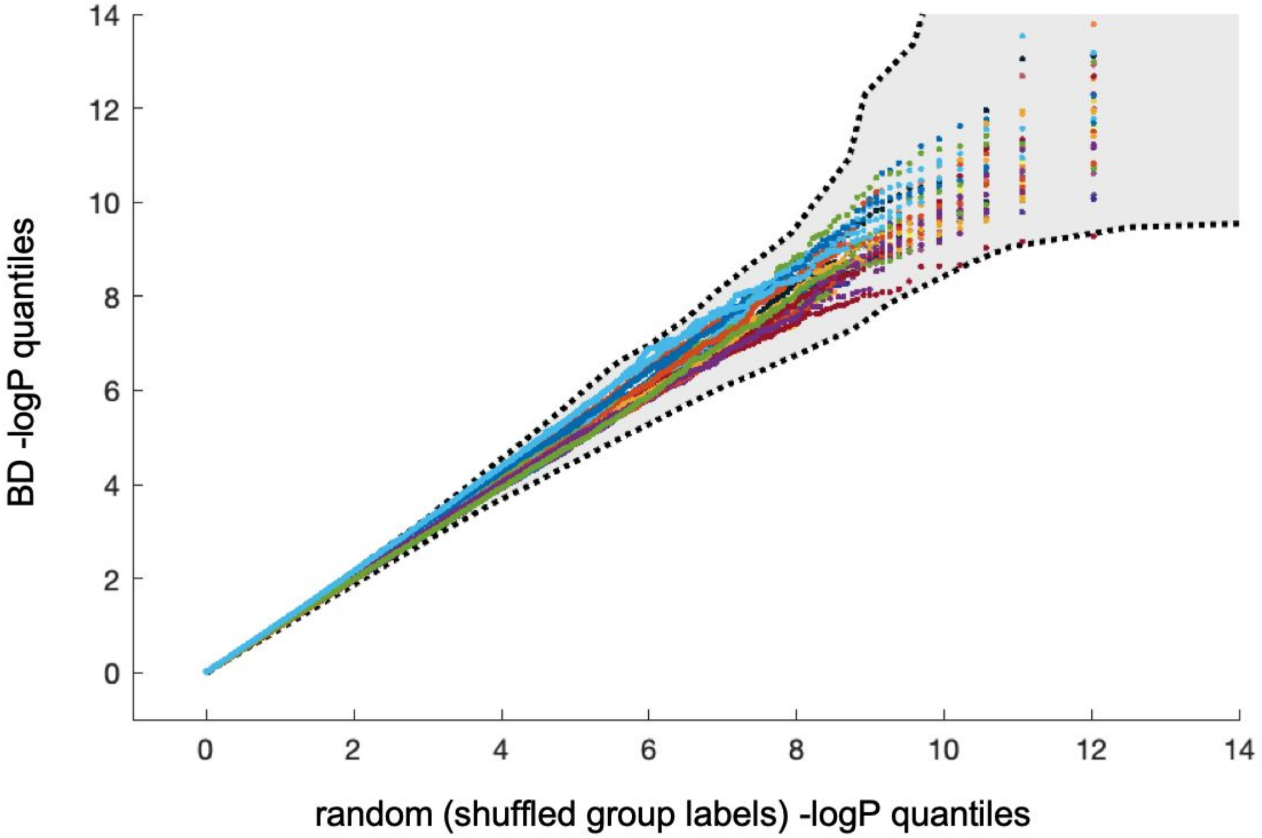

Figure S12. Q-Q plot of BD1-33 with BDgene-residing chromosome removed before p-value quantiles were computed. 100 random vs random Q-Q plots (gray area) with their outline (dashed lines) displayed.

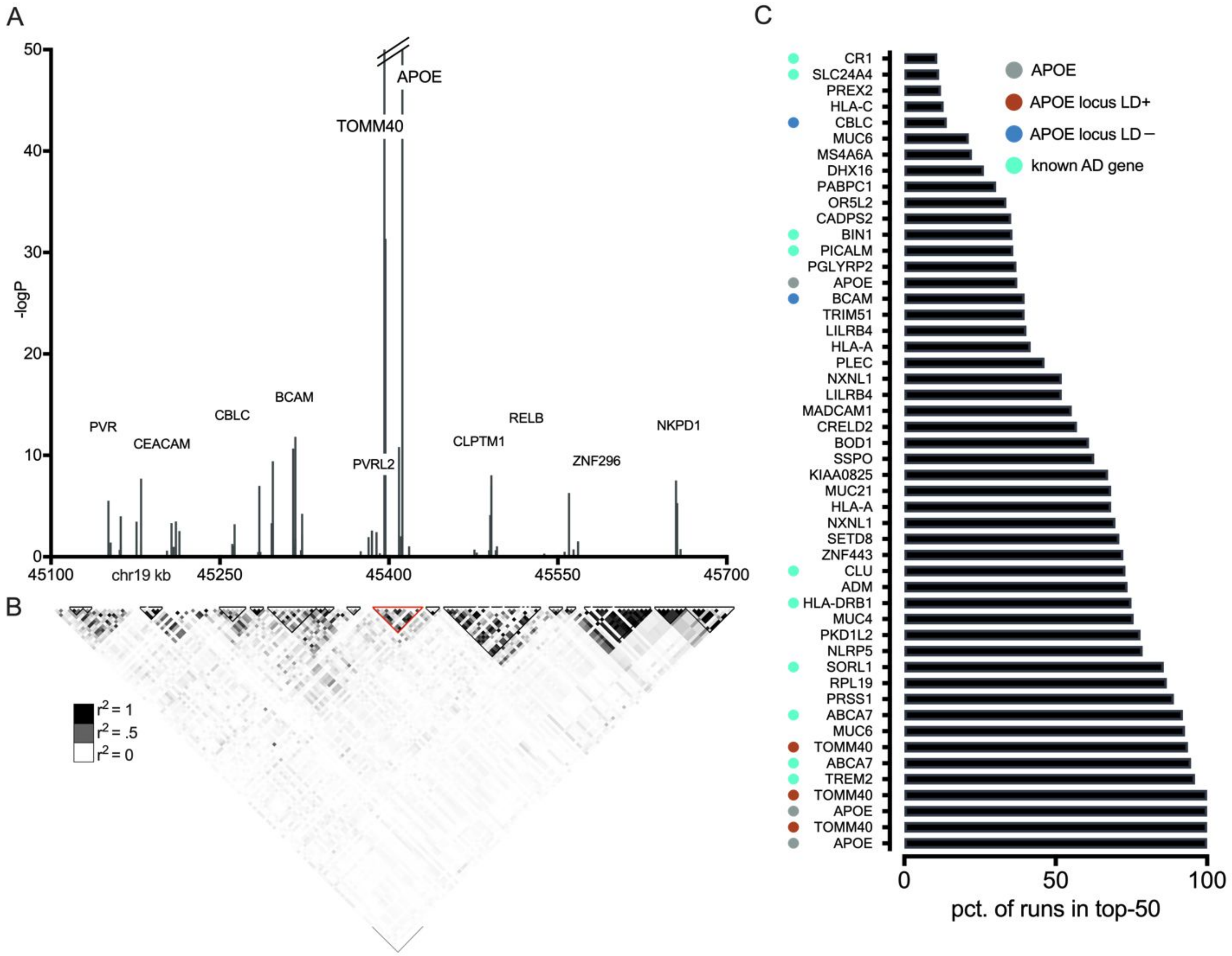

Figure S13: SNPs in linkage disequilibrium with APOE (other than TOMM40) are not included as training features. (A) Displays -log p-values for Fisher's exact test of case:ctrl asymmetry at each SNP within 250 kb of the APOE gene. (B) Haploview plot of LD structure extended APOE locus. The red triangle includes loci that span APOE, TOMM40, and PVRL2 genes. SNPs within these genes display linkage with each other. SNPs in this block are not statistically linked with SNPs in other genes in this region (according to hapmap phase-3 CEU data). (C) Each run of netSNP uses a randomly generated training set, which in turn yield a variety of top-50 SNPs - the 50 SNPs with the lowest p-values for a given run. This plot displays the 50 SNPs occurring most often among the set of 50 SNPs with the lowest p-values; each horizontal bar indicates how often. APOE locus LD+/- indicates SNPs within 250 kb of APOE that do (+) or do not (-) display linkage disequilibrium with APOE.

Table S1. netSNP identified tSNPs with greatest absolute average ***CVt***
when APOE locus variants were excluded from the training set

tSNPs predicted to confer most protection against AD

Chr	Pos	Gene	***mCVt***	FishP	MAF
19	14,044,067	PODNL1	**-0.2264**	1.0E-02	0.001
19	45,412,079	APOE	**-0.22537**	7.1E-38	0.079
5	612,536	CEP72	**-0.21151**	5.7E-03	0.002
6	1,390,303	FOXF2	**-0.20768**	1.4E-02	0.003
12	88,472,911	CEP290	**-0.19076**	8.1E-03	0.004
8	144,991,347	PLEC	**-0.18896**	9.0E-03	0.003
12	132,400,547	ULK1	**-0.18757**	2.8E-02	0.002
17	39,623,282	KRT32	**-0.18671**	1.6E-02	0.003
1	154,988,966	ZBTB7B	**-0.18046**	3.5E-03	0.002
3	41,497,081	ULK4	**-0.17573**	1.6E-02	0.002
1	16,890,642	NBPF1	**-0.17167**	3.3E-02	0.002
8	144,992,781	PLEC	**-0.17057**	5.5E-03	0.002
11	57,467,411	ZDHHC5	**-0.16888**	3.5E-03	0.002
1	15,642,964	FHAD1	**-0.15989**	1.7E-02	0.004
11	1,017,294	MUC6	**-0.15614**	1.4E-07	0.01
16	87,760,445	KLHDC4	**-0.1549**	3.4E-02	0.001
9	139,396,933	NOTCH1	**-0.15486**	3.3E-02	0.004
5	95,249,572	ELL2	**-0.15458**	7.6E-03	0.002
2	26,533,898	GPR113	**-0.15336**	1.4E-03	0.005
12	69,113,184	NUP107	**-0.1488**	5.6E-03	0.006
6	36,343,720	ETV7	**-0.14852**	5.5E-03	0.005
1	43,296,522	ERMAP	**-0.14657**	2.0E-02	0.004
19	18,547,486	ISYNA1	**-0.14422**	6.7E-03	0.005
13	52,346,022	DHRS12	**-0.14361**	2.4E-03	0.001
13	45,589,616	GPALPP1	**-0.14182**	1.4E-04	0.006

Rows 26:1000 available online

* Previously published AD-linked gene

tSNPs predicted to confer the most risk for AD

Chr	Pos	Gene	***mCVt***	FishP	MAF
11	10,327,875	ADM	**0.297984**	4.1E-08	0.008
19	45,411,941	APOE	**0.28784**	3.4E-111	0.135
11	1,085,798	MUC2	**0.222727**	1.5E-02	0.002
19	45,396,144	TOMM40	**0.222033**	7.6E-56	0.134
17	33,329,814	LIG3	**0.219692**	1.5E-02	0.002
7	23,213,734	KLHL7	**0.205186**	5.1E-03	0.003
9	105,767,074	CYLC2	**0.202819**	1.1E-02	0.002
9	96,070,669	WNK2	**0.201183**	1.1E-02	0.003
13	73,357,758	PIBF1	**0.197754**	1.6E-02	0.002
11	5,566,076	OR52H1	**0.19524**	3.1E-03	0.004
15	41,862,356	TYRO3	**0.194049**	8.4E-18	0.018
2	201,478,556	AOX1	**0.193213**	1.1E-02	0.003
20	37,258,198	ARHGAP40	**0.192566**	1.1E-02	0.002
10	120,907,358	SFXN4	**0.190474**	4.9E-05	0.002
14	72,090,863	SIPA1L1	**0.189259**	1.5E-02	0.004
19	7,505,163	ARHGEF18	**0.187715**	2.6E-03	0.005
19	45,322,671	BCAM	**0.186076**	1.5E-02	0.002
1	201,263,108	PKP1	**0.184712**	2.6E-03	0.002
16	16,218,641	ABCC1	**0.183192**	6.6E-03	0.002
3	50,293,616	GNAI2	**0.183059**	1.7E-03	0.003
13	111,142,112	COL4A2	**0.18252**	1.0E-02	0.004
15	25,963,545	ATP10A	**0.182421**	1.2E-02	0.002
1	113,126,734	ST7L	**0.182378**	1.9E-02	0.002
12	97,136,336	C12orf55	**0.182093**	1.1E-02	0.002
20	464,604	CSNK2A1	**0.182092**	1.9E-02	0.001

Rows 26:1000 available online

* Previously published AD-linked gene

Table S2. netSNP identified tSNPs with greatest absolute average ***CVt*** when the test set was assigned the *APOE* ε4 allele

Chr	Pos	Gene	***mCVt***	FishP	MAF
19	45,412,079	APOE ε2*	**-0.041**	2.73E-65	0.074
16	22,285,007	EEF2K	**-0.041**	3.19E-02	0.005
14	94,528,876	DDX24	**-0.037**	1.60E-02	0.006
20	210,048	DEFB129	**-0.036**	3.73E-02	0.003
12	88,472,911	CEP290	**-0.031**	2.14E-03	0.004
12	69,113,184	NUP107	**-0.002**	3.26E-04	0.006
21	44,839,263	SIK1	**-0.001**	4.27E-02	0.008
11	1,017,294	MUC6	**0.000**	3.54E-04	0.01
22	28,194,912	MN1	**0.003**	4.71E-02	0.005
19	41,173,871	NUMBL	**0.003**	7.70E-02	0.013
11	47,587,390	PTPMT1	**0.005**	1.88E-02	0.003
9	133,759,378	ABL1	**0.008**	1.04E-03	0.004
7	100,275,444	GNB2	**0.009**	3.54E-03	0.004
12	54,963,142	PDE1B	**0.011**	6.45E-02	0.004
6	36,343,720	ETV7	**0.018**	9.10E-03	0.005
19	1,881,453	ABHD17A	**0.020**	8.91E-03	0.009
19	18,547,486	ISYNA1	**0.040**	9.04E-04	0.004
11	62,543,326	TAF6L	**0.041**	2.37E-04	0.008
14	50,844,256	CDKL1	**0.049**	1.16E-06	0.031
10	61,833,414	ANK3	**0.050**	3.95E-04	0.006
7	149,473,614	SSPO	**0.062**	2.92E-05	0.028

* Previously published AD-linked gene

			'Bad Disease (BD)' construction APOE2-like & APOE4-like genomic loci						Correlation with Disease Age-of-Onset			
Gene	Disease	Chr	APOE2 Pos	APOE2 AD Odds Ratio	APOE2 MAF	APOE4 Pos	APOE4 AD Odds Ratio	APOE4 MAF	*Protective* ***tSNP*** *P*	Protective Weighted-***tSNP*** P	*Risk* ***tSNP*** P	Risk Weighted-***tSNP*** P
APOE	AD	19	45412079	2.41	0.076	45411941	0.30	0.147	2.0E-04	1.7E-85	2.0E-05	2.5E-86
CHSY1	BD1	15	101718700	0.97	0.080	101717888	1.01	0.147	0.647	0.657	0.183	0.437
ABCB4	BD2	7	87056176	0.98	0.077	87092185	1.00	0.148	0.140	0.934	0.157	0.872
DMKN	BD3	19	36003962	0.99	0.072	36004171	1.04	0.144	0.169	0.756	0.758	0.240
DSG1	BD4	18	28919779	0.97	0.075	28934681	0.95	0.149	0.011	0.842	0.819	0.138
FNDC7	BD5	1	109261509	1.04	0.077	109268441	1.02	0.146	0.285	0.146	0.121	0.153
KRT5	BD6	12	52913668	1.00	0.076	52912870	1.03	0.143	0.342	0.690	0.777	0.404
SCEL	BD7	13	78178550	1.00	0.072	78216915	1.00	0.147	0.382	0.272	0.862	0.129
SCUBE2	BD8	11	9051475	1.04	0.078	9069046	1.00	0.144	0.078	0.484	0.872	0.786
SOAT1	BD9	1	179319541	0.96	0.072	179312752	1.00	0.148	0.963	0.052	0.585	0.071
TIAM2	BD10	6	155569168	0.99	0.079	155486519	1.00	0.144	0.581	0.096	0.163	0.075
TNS3	BD11	7	47343083	1.07	0.072	47436497	1.04	0.150	0.141	0.231	0.106	0.339
WNK1	BD12	12	971291	1.02	0.073	994014	1.00	0.144	0.099	0.782	0.241	0.405

Table S3. netSNP validation results

Left columns (1-9), summary data on 12 existing genes containing two loci that have population frequency (MAF) almost identical to those of APOE2 ('APOE2-like') and APOE4 (APOE4-like). Simulated diseases ('bad disease', BD) were assigned to individuals (including age of diagnosis) with APOE2-like and APOE4-like SNPs based on the prevalence (and age of onset) of AD for APOE2 and APOE4 (See Figure S7). Right columns (10-13) are probability values testing the correlation between number (columns 10, 12) or CVt-weighted number (columns 11, 13) of netSNP-identified SNPs and age of disease onset. The netSNP procedure performed for BD exactly as for AD. Note that no correlation is significant (alpha=0.01) for any BD; while netSNP-identified SNPs (top row) display strong correlation with AD age of onset.
